# Exercise improves choroid plexus epithelial cells metabolism to prevent glial cell-associated neurodegeneration

**DOI:** 10.3389/fphar.2022.1010785

**Published:** 2022-09-16

**Authors:** Yisheng Chen, Zhiwen Luo, Yaying Sun, Fangqi Li, Zhihua Han, Beijie Qi, Jinrong Lin, Wei-Wei Lin, Mengxuan Yao, Xueran Kang, Jiebin Huang, Chenyu Sun, Chenting Ying, Chenyang Guo, Yuzhen Xu, Jiwu Chen, Shiyi Chen

**Affiliations:** ^1^ Huashan Hospital, Fudan University, Shanghai, China; ^2^ Department of Orthopedics, Shanghai General Hospital, Shanghai Jiao Tong University School of Medicine, Shanghai Jiao Tong University, Shanghai, China; ^3^ Department of Neurosurgery, Second Affiliated Hospital of Zhejiang University School of Medicine, Zhejiang University, Hangzhou, China; ^4^ Department of Orthopaedic Surgery, The Third Hospital of Hebei Medical University, Shijiazhuang, Hebei; ^5^ Shanghai Jiao Tong University School of Medicine, Shanghai Jiao Tong University, Shanghai, Hebei; ^6^ Ruijin Hospital, Shanghai Jiao Tong University School of Medicine, Shanghai, China; ^7^ AMITA Health Saint Joseph Hospital Chicago, Chicago, IL, United States; ^8^ Department of Orthopaedics, The Second Affiliated Hospital, Zhejiang University School of Medicine, Hangzhou, China; ^9^ Department of Rehabilitation, The Second Affiliated Hospital of Shandong First Medical University, Taian, China

**Keywords:** Alzheimer’s disease, astrocytes, brain energy metabolism, choroid plexus epithelial cells, exercise, multicomics, pharmacology

## Abstract

Recent studies have shown that physical activities can prevent aging-related neurodegeneration. Exercise improves the metabolic landscape of the body. However, the role of these differential metabolites in preventing neurovascular unit degeneration (NVU) is still unclear. Here, we performed single-cell analysis of brain tissue from young and old mice. Normalized mutual information (NMI) was used to measure heterogeneity between each pair of cells using the non-negative Matrix Factorization (NMF) method. Astrocytes and choroid plexus epithelial cells (CPC), two types of CNS glial cells, differed significantly in heterogeneity depending on their aging status and intercellular interactions. The MetaboAnalyst 5.0 database and the scMetabolism package were used to analyze and calculate the differential metabolic pathways associated with aging in the CPC. These mRNAs and corresponding proteins were involved in the metabolites (R)-3-Hydroxybutyric acid, 2-Hydroxyglutarate, 2-Ketobutyric acid, 3-Hydroxyanthranilic acid, Fumaric acid, L-Leucine, and Oxidized glutathione pathways in CPC. Our results showed that CPC age heterogeneity-associated proteins (ECHS1, GSTT1, HSD17B10, LDHA, and LDHB) might be directly targeted by the metabolite of oxidized glutathione (GSSG). Further molecular dynamics and free-energy simulations confirmed the insight into GSSG’s targeting function and free-energy barrier on these CPC age heterogeneity-associated proteins. By inhibiting these proteins in CPC, GSSG inhibits brain energy metabolism, whereas exercise improves the metabolic pathway activity of CPC in NVU by regulating GSSG homeostasis. In order to develop drugs targeting neurodegenerative diseases, further studies are needed to understand how physical exercise enhances NVU function and metabolism by modulating CPC-glial cell interactions.

## Introduction

The number of people living with dementia worldwide has doubled since 1990, from nearly 21 million to 44 million (Nichols et al., 2019). Alzheimer’s disease and associated dementia (ADRD) is a general term for irreversible and progressive neuronal damage. ADRD is a common symptom of various neurodegenerative diseases and one of the major causes of disability and death in the elderly (Gonzales et al., 2022). Even in older adults without dementia, cognitive decline and neurodegenerative changes with age are evident even in those without a history of dementia, suggesting a common pathophysiological mechanism for ADRD (Gonzales et al., 2022). With age, brain glucose metabolism deteriorates, leading to problems with energy supply. In AD, glucose metabolism is perturbed even before symptoms appear (Cunnane et al., 2020). Dysregulation of synaptic signaling and ion transport across membranes is caused by impaired brain glucose metabolism (Cunnane et al., 2020). Cerebral glucose hypometabolism in ADRD-related neurodegenerative diseases results from various complex causes, including impaired neuronal glucose uptake, impairment of aerobic glycolysis, impaired tricarboxylic acid cycle, dysfunctional axonal transport, and lack of glial cell energy supply (Cunnane et al., 2020; Li et al., 2022a). In recent years, scientists have been exploring therapeutic strategies to combat ADRD by improving brain energy metabolism (Cunnane et al., 2020; Scariot et al., 2021). Previous studies have shown that exercise may improve brain energy metabolism by improving growth and development, the body’s ability to work, and the overall fitness of the individual (Camandola and Mattson, 2017; Mattson and Arumugam, 2018; Okamoto et al., 2021; Li et al., 2022a).

Exercise increases blood flow to the brain and improves macroscopic hemodynamics and microscopic neurovascular functions (Kosmala et al., 2013; Yuan et al., 2015; Seidel et al., 2019; Hafez et al., 2020; Burma et al., 2021). Numerous studies have shown that exercise has a protective effect on cerebral vascular function as it protects the blood-brain barrier, promotes the formation of new blood vessels, and reduces neuronal apoptosis, contributing to improved neurological function after cerebral ischemia (Hafez et al., 2021; Zhang et al., 2022a). Cerebrovascular disease-related ADRD could be improved with exercise (Nation et al., 2011; McGough et al., 2017). Research has also shown that exercise interventions can prevent ADRD and stroke-related neurovascular disease in the aging brain (Lucas et al., 2015). With advances in neuroscience research, neurovascular unit injury has gained increasing attention as a contributing factor to ADRD pathogenesis (Liu et al., 2019). A neurovascular unit (NVU) comprises neurons, astrocytes, microglia, vascular endothelial cells, perivascular cells, basement membrane, and extracellular matrix. We speculated that exercise prevents AD by improving NVU function (McGough et al., 2017).

Exercise improves the metabolic landscape of vascular cells and prevents diseases associated with vascular dynamics (Teuwen et al., 2019; Wang et al., 2019; Beckman et al., 2020; Hasan and Fischer, 2022; Khoramipour et al., 2022). Metabolites have a neurovascular remodeling function in humans that is critical to brain function (Smith and Ainslie, 2017; Mirzahosseini et al., 2022). In recent studies, small molecule metabolites have been shown to target key enzymes in the NVU microenvironment to improve the treatment of neurological diseases via metabolically targeted interventions (Wang et al., 2021; Mirzahosseini et al., 2022). It has been shown that the endogenous ketone body beta-hydroxybutyrate facilitates the recovery of peri-infarct neurovascular function and metabolism (Bazzigaluppi et al., 2018). In contrast to traditional ab initio drug design, molecular dynamics simulation (MDS) can accurately predict the binding patterns of small molecule metabolites to target proteins (Aci-Sèche et al., 2016; Al-Qattan et al., 2018; Do et al., 2018). We propose to use MDS to identify potential patterns of metabolite targeting of key proteins that may shed light on how exercise improves cerebrovascular metabolism (Do et al., 2018; Liu et al., 2018; Pradiba et al., 2018Pradiba et al., 2018).

Exercise prescription improves the metabolic profile of the body’s microenvironment, and these metabolites prevent neurodegeneration by targeting NVUs. However, the mechanism behind this is not well understood. To correct the abnormal microenvironment in neurodegenerative diseases, we use a single-cell and bioinformatic approach to identify key cellular subtypes and metabolic pathways involved in the development of neurodegenerative diseases. We also aim to conduct pharmaceutical studies and develop drugs to treat neurodegenerative diseases.

## Methods

### Data acquisition

Datasets were downloaded from Gene Expression Omnibus (GEO, http://www.ncbi.nlm.nih.gov/GEO/) (Barrett et al., 2012). The keywords “single-cell”, “blood-brain barrier”, and “aged” were used to retrieve age-related studies and scRNA-seq transcriptome datasets in the GEO database. The GSE147693 dataset included single-cell transcriptome sequence data from young and old cerebrovascular cells with NVU, with 3 replicates of each group (Zhao et al., 2020). Two groups of male mice were included in this dataset: those 2–3 months old and 18–20 months old. As described in previous studies, there were 63,300 cells in the single-cell master count matrix [http://cells.ucsc.edu/?ds=aging-brain]. This dataset can be visualized, and is also available online [https://singlecell.broadinstitute. org/single_cell/study/SCP829/aging-mouse-brain-kolab]. Our study examined the relationship between age and the transcriptional profile of NVU using the GSE147693 dataset.

### Analyzing metabolic pathways associated with exercise

Koay YC et al. identified metabolites that differ in circulating blood after exercise in their study. They demonstrated the effects of regular exercise, which could be used as a new treatment strategy for metabolic conditions associated with vascular disease (Koay et al., 2021). Supplementary files from this study showed metabolic adaptation to an 80-days exercise intervention after regulating the lifestyle factors like diet, sleep, and physical activity. Exercise-regulated metabolites were explored in this study. The metabolic substances regulated by physical exercise were identified in this study.

The MetaboAnalyst database, a comprehensive suite of web-based tools, was used in this study for metabolomics data analysis, visualization, and functional annotation (Chong et al., 2019). MetaboAnalyst results were further visualized using Hiplot, a free web service (Li et al., 2022b).

### scRNA-seq quality filtering, dimensionality reduction, and clustering

ScRNA-seq data from the GSE147693 dataset were analyzed using the “Seurat” package for cellular integration, dimensionality reduction, clustering, and cellular annotation (Stuart et al., 2019; Lin et al., 2021; Chen et al., 2022a). Data from scRNA-seq was first checked for quality. Quality control criteria were as follows: 1. Genes expressed in fewer than three cells were removed, leaving a total of 19,746 genes; 2. Cells containing less than 400 genes or more than 12,000 genes were removed; 3. Cells containing more than 10 unique molecular identifiers (UMIs) from the mitochondrial genome were removed; 4. Cells expressing more than 5 hemoglobin-related genes were removed; 5. Gene features expressed by no more than 10 cells were excluded. Finally, 44,860 cells and 19,029 gene signatures were obtained. With Seurat, the canonical correlation analysis (CCA)-based integration function was used to eliminate batch effects. The number of clusters was determined using the “FindNeighbors” and “FindClusters” functions, with a resolution value of 0.6. Seurat’s FindVariableFeatures function was used to identify genes with a high variance. For further clustering analysis, UMAP was applied to the cells following the “RunUMAP” command (Becht et al., 2019). The final cell annotation was then completed using the cell lineage marker genes applied previously (Zhao et al., 2020). Proportions of each cell type in the two groups were visualized using the “ggplot2” package. Seurat’s “FindMarker” function was also used to extract target cells from the scRNA-seq data. Differentially expressed genes (DEGs) were calculated between the older and younger groups at the single cell level. |log FC| greater than 0.25 and *p*-value less than 0.05 were considered statistically significant differences.

### Non-negative matrix factorization clustering

NMF is a soft clustering method that extracts features in matrices well without needing *a priori* knowledge. By further analyzing the features, NMF can give the probability of a sample belonging to a particular class and is particularly useful for analyzing continuous developmental processes in a single cell. Combining this approach with traditional clustering analysis allows defining more complex sets of cell states and corresponding gene features. This study used Consensus Non-negative Matrix factorization (cNMF) to solve the problem of not having unique results after decomposing NMF. High variance genes derived from the above analysis were selected. Hyperparameters were selected based on the following: 1. Firstly, the lithotripsy map of PCA and the diagnostic map given by the authors were combined to determine the components that should be selected; 2. The components with extreme distributions were removed after combining with the cell score distribution of the Usage matrix. Finally, the cosine distances were calculated on NMF, and the K-NearestNeighbor (kNN) was used to stabilize the optimization map and perform the clustering analysis.

### Estimating heterogeneity

Previous studies have examined the heterogeneity of single cell profiles in the mouse brain vasculature by examining the average Normalized Mutual Information (NMI) between different cell types (Marjanovic et al., 2020). Each subtype cluster consisted of 100 differentially expressed genes, whose expression was discretized by equal width intervals. The median NMI of the sampled pairs within each time point was calculated by sampling 100 cells from each class 100 times and accounting for differences in the number of cells between samples. NMI was calculated between each pair of cells. *p*-value was calculated for the difference in NMI value between the two groups by comparing the number of sub-samples in the A group to those in the B group.

### Cell-cell communication analysis

Cell-cell communication was analyzed to identify cellular interactions across cell subtypes and other cells in the cerebral vasculature at different ages (Kumar et al., 2018; Maia et al., 2018; Shao et al., 2020; Chen et al., 2022b). CellChat was used to infer ligand-receptor crosstalk between single cells (Jin et al., 2021). Identified ligands or receptors were then projected onto a network of protein-protein interactions, and a permutation test was performed to infer biologically meaningful cell-cell communication. Intercellular communication network senders, receivers, mediators, and influencers were also identified with CellChat. “CellChat”, “Seurat”, “ggplot2”, and “ggalluvial” were used for statistical analysis and mapping.

### Single-cell metabolic analysis

Metabolic activity was quantified in single cells using scMetabolism, a software developed by Fudan University Institute, to implement the scRNA-seq metabolic analysis. Based on a conventional single-cell matrix file, the software utilizes the vision algorithm to determine the activity score of each cell in every metabolic pathway (Wu et al., 2022). scMetabolism software was pre-populated with 85 KEGG pathways and 82 Reactome entries. The metabolic activity was analyzed after transforming the altered data set homologously. And the metabolic score was calculated by the Vision algorithm (DeTomaso et al., 2019). Finally, the metabolic activity of various pathways among different groups was determined to obtain pathways with significant differences (McDavid et al., 2013).

### CytoTRACE analysis

CytoTRACE is a computational method that predicts cell direction and differentiation status from single-cell RNA sequencing (Gulati et al., 2020). CytoTRACE predicts differentiation status in scRNA-seq data without any *a priori* knowledge. CytoTRACE captures gene count features by summing the total number of genes expressed greater than zero in every cell. By exploiting local similarity between cells and applying a two-step smoothing procedure, the estimation of the GCS vector was improved iteratively. Finally, the graphs were saved with the “plotCytoTRACE” code.

### Gene ontology functional enrichment analysis and kyoto encyclopedia of genes and genomes pathway analysis

As in previous studies, GO enrichment analysis was performed using the molecular function (MF), biological processes (BP), and cellular components (CC) (Chen et al., 2017; Chen et al., 2022b; Zhang et al., 2022b; Zhang et al., 2022c; Feng et al., 2022; Kang et al., 2022). To determine the best functional and *in vivo* pathways significantly enriched by the active ingredient targets, the significance of the KEGG pathway was set at *p* < 0.05. ClusterProfiler and ggplot2 in R were used to plot bar graphs of the GO and KEGG pathways (McDavid et al., 2013; DeTomaso et al., 2019).

### Docking of small molecules and proteins

ECHS1 (P30084), GSTT1 (P30711), HSD17B10 (Q99714), LDHA (P00338), and LDHB (P07195) Protein Data Bank (PDB) files were obtained from the uniport database (https://www.uniprot.org/uniprot) (Cramer, 2021). Quercetin quantum chemical optimization, including correction of bond length, bond angle, and dihedral angle, and calculation of RESP2.0 fixed charge, was carried out using Quantum Chemical Software, Orca. (Neese et al., 2020). Using the software smina, docking of the ligand with hydrogen was performed on the protonated protein. The lowest energy conformation was selected as the final conformation for kinetic simulation (Masters et al., 2020).

### Molecular dynamics simulation

GROMACS 2019.4 software was used to perform MD simulations, Amber14sb was chosen as the protein force field, Gaff2 was chosen for small molecules, and the TIP3P water model was used to build a water box and add sodium ions to balance the complex system. Particle-mesh Ewald (PME) uses the steepest descent method for energy minimization of the maximum number of steps (50,000) in the elastic simulations by Verlet and CG algorithms, respectively (Van Der Spoel et al., 2005). The Coulomb force cutoff distance and van der Waals radius cutoff distance were both 1.4 nm, and the system was equilibrated using the regular system (NVT) and isothermal isobaric system (NPT). The MD simulation was performed for 100 ns at room temperature and pressure. The LINCS algorithm constrained the hydrogen bonds with a 2 fs integration step in MD simulations. Particle-mesh Ewald (PME) was calculated with 1.2 nm as the cutoff value. NVT and NPT equilibrium simulations were conducted at 300 K for 30 ps, and the MD simulations for the protein-ligand complex were performed for 100 ns. In order to evaluate the tightness of the system structure, the radius of rotation (Rg) was used.

### Calculation of free energy of binding between proteins and metabolites

Molecular mechanics [MM] with Poisson--Boltzmann [PB] and surface area solvation (MM/GBSA) method was used to calculate the free binding energy between receptor and ligands (Valdés-Tresanco et al., 2021). The equilibrium MD trajectory (20–30 ns) was calculated using the following equation:

$${\Delta G}_{bind}={\Delta G}_{complex}\;–\;\left({\Delta G}_{receptor}+{\Delta G}_{ligand}\right)$$

$$={\Delta E}_{internal}+{\Delta E}_{VDW}+{\Delta E}_{elec}{+\Delta G}_{GB}+{\Delta G}_{SA}$$

The above equation represents internal energy, van der Waals interaction, and electrostatic interactions. The internal energies included Ebond, Eangle, and Etorsion, collectively referred to as the free energy of solvation. GGB is the polar solvation free energy, and GSA is the non-polar solvation free energy. For this paper, the GB model developed by Nguyen et al. was used for the calculation (igb = 8). The non-polar solvation free energy (GSA) is calculated based on the product of surface tension (γ) and solvent accessible surface area (SA), GSA = 0.0072 × SASA (Weiser et al., 1999).

### Statistical analysis

R software version 4.1.1 was used to create all plots, and the Venn diagrams were drawn using the “VennDiagram” R package. Chi-square tests were used to compare the proportions of cell types between the two groups, and *p* < 0.05 was considered statistically significant.

## Results

### Single-cell analysis and clustering of brain tissue in young and old mice

**FIGURE 1:**
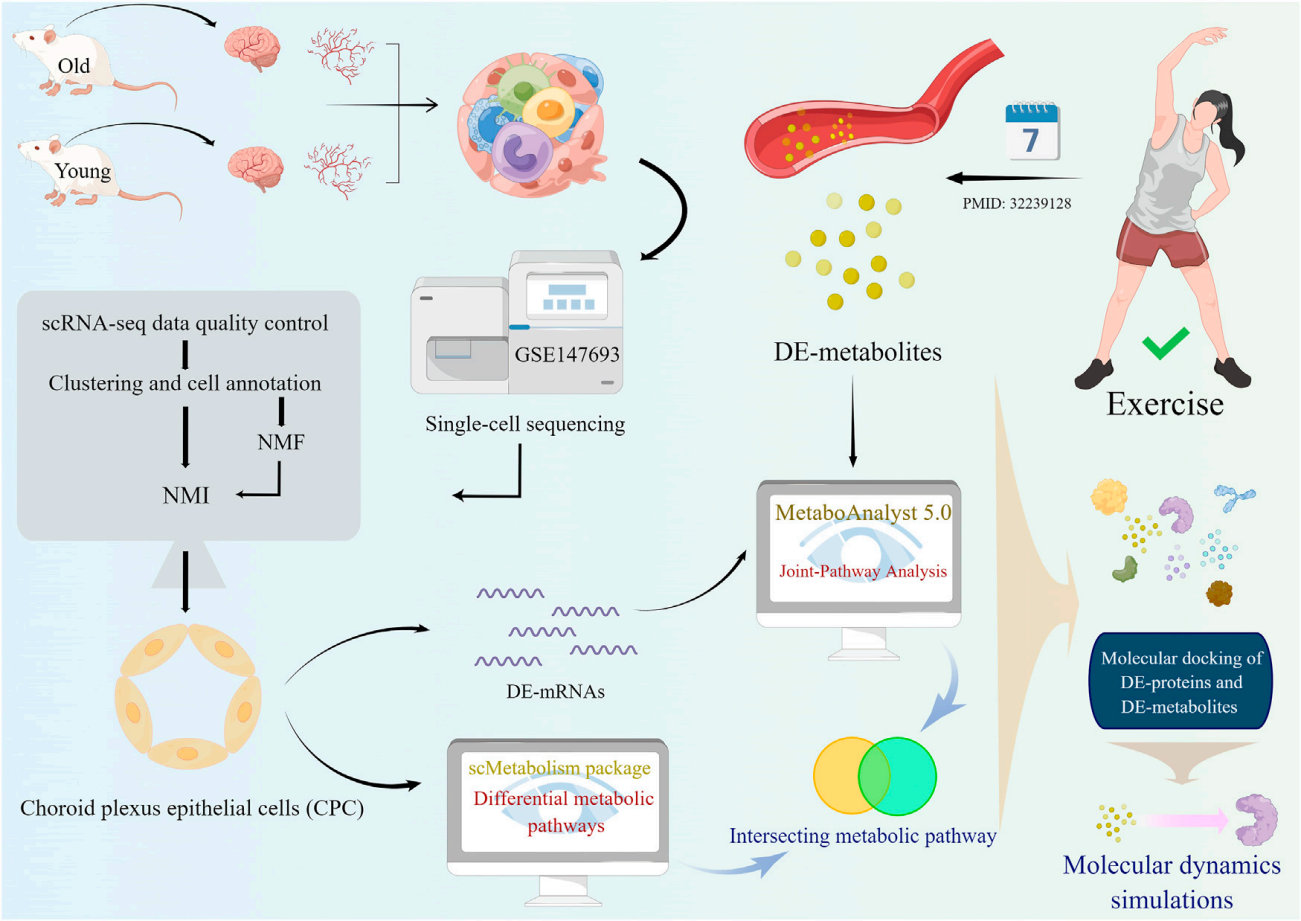
A schematic diagram showing the study flow. Firstly, single-cell analysis was performed on young and old mouse brain tissues from the GSE147693 dataset. Then the non-negative matrix factorization (NMF) method was used to measure heterogeneity between each pair of cells. Among the two types of glial cells in the CNS, astrocytes and choroid plexus epithelial cells (CPC), the heterogeneity of astrocytes changes more markedly with age and there are several interesting intercellular interactions. Age-related differential metabolic pathways in CPC were analyzed and calculated using the scMetabolism package. Based on molecular dynamics simulations, oxidized glutathione targets CPC proteins that exhibit age heterogeneity.

**FIGURE 2:**
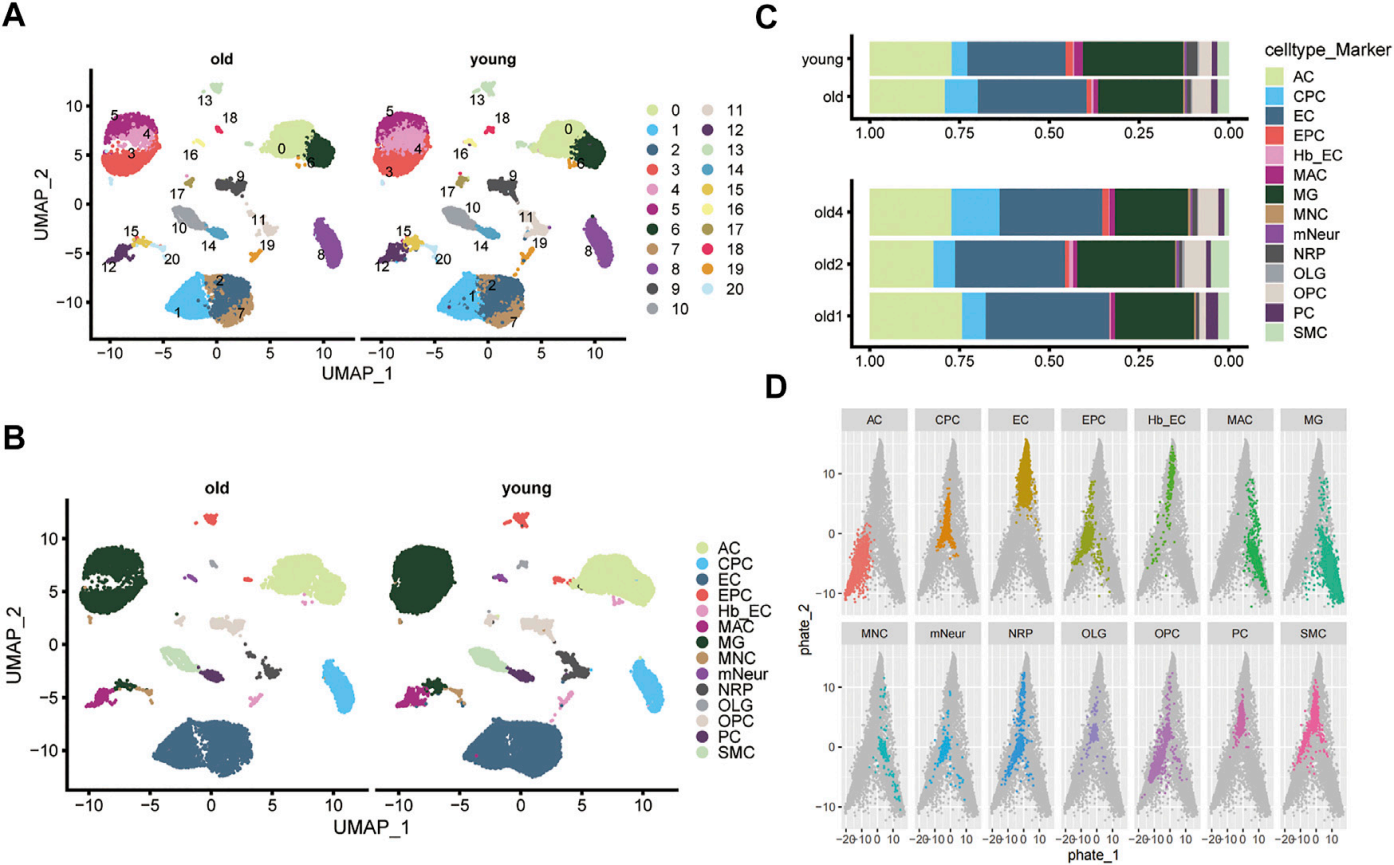
Single-cell clustering and sub-clustering of mouse brain tissue. **(A)** Uniform Manifold Approximation and Projection (UMAP) plot shows the clustering of cells in the old and young groups. Different colors distinguish different subgroups. **(B)** The UMAP plot shows the 14 cell types identified in the old and young groups (including: AC, CPC, EC, EPC, hb EC, MAC, MG, MNC, mNeur, NRP, OLG, OPC, PC, and SMC). **(C)** The bar graphs show the proportion of each cell type in the old and young groups. **(D)** PHATE maps the distribution of different cell types based on the scRNA-seq profile of GSE147693. The colored dots indicate the specified cell types, and gray dots represent other cells.

Figure 1 shows the flow chart for this study. First, mouse cerebrovascular tissues from the GSE147693 dataset were subjected to single-cell analysis and quality control (Supplementary Figure S1A,B). A total of 44,860 cells and 19,029 genetic features were obtained after removing cells that did not meet the inclusion criteria (see Methods). UMAP plots clustered cells in the older and younger groups (Figure 2A). Koay YC et al. identified 14 cell types in old and young groups, including astrocytes (AC), choroid plexus epithelial cells (CPC), brain endothelial cell (EC), endothelial progenitor cells (EPC), endothelial cells (EC), macrophages (MAC), microglia (MG), midbrain neuron cells (MNC), mature neuronal cells (mNeur), neuron-restricted precursor cells (NRP), oligodendroglia cells (OLG), oligodendrocyte precursor cells (OPC), pericytes (PC), and smooth muscle cells (SMC, Figure 2B) (Koay et al., 2021). Each cell cluster’s top ten marker genes were listed (Supplementary Figure S2). The bar graphs show the proportion of each cell type in the old and young groups (Figure 2C). We analyzed and clustered the single brain cells based on those information. Finally, we mapped the distribution of different cell types and saved the relevant parameters as NMF profiles (Figure 2D).

### Differences between older and younger groups in choroid plexus epithelial cells

**FIGURE 3:**
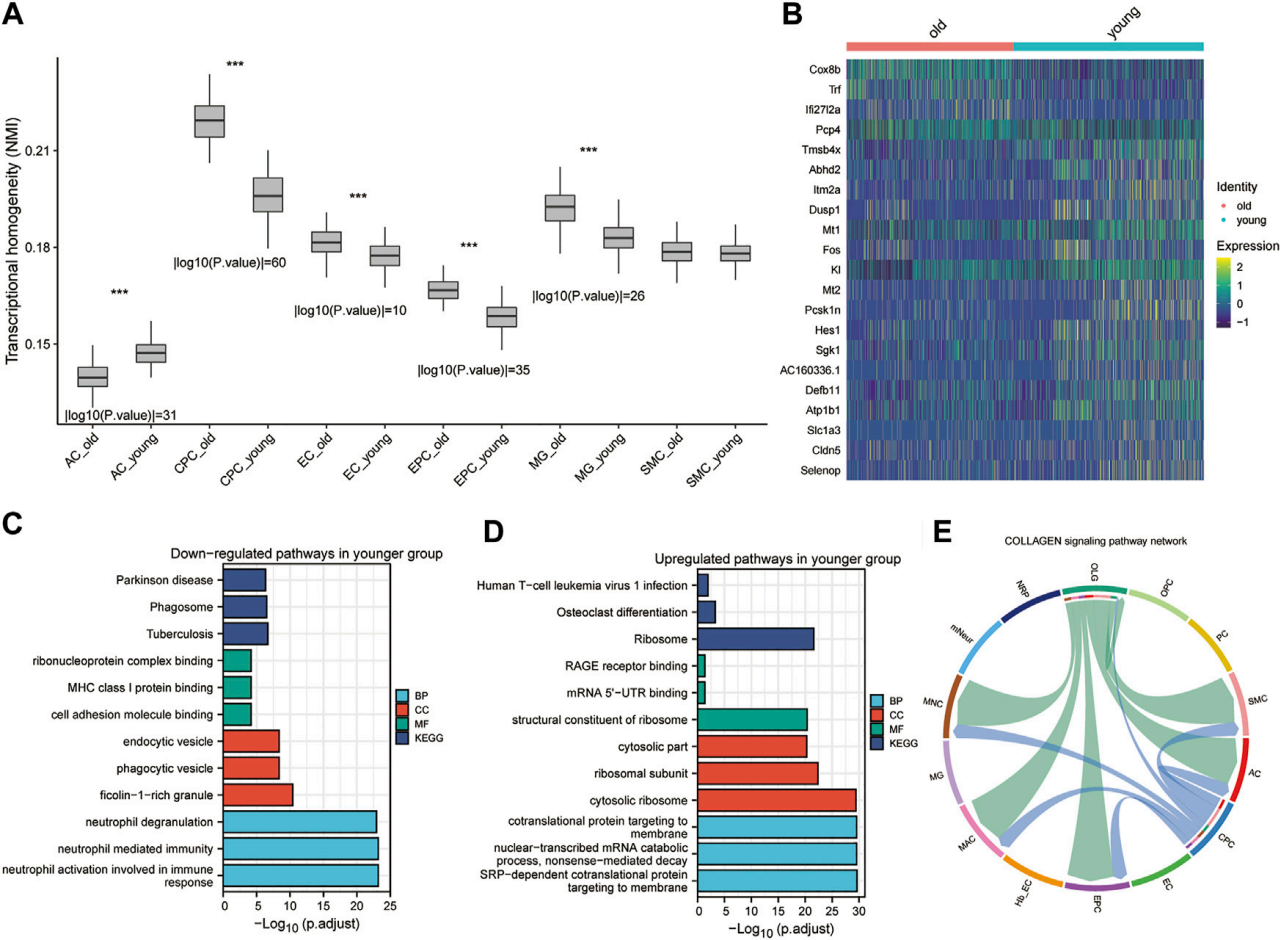
Heterogeneity and differential analysis of single cells in mouse brain tissue. **(A)**. Box plot showing transcriptional heterogeneity between older and younger cells for each cell subtype using Normalized Mutual Information (NMI). * *p* < 0.05, ** *p* < 0.01, *** *p* < 0.001. **(B)** The heatmap shows the main DEGs in CPC obtained by applying the “FindVariableFeatures” function. **(C,D)** Downregulated and upregulated GO **(C)** and KEGG pathways **(D)** in the younger group. **(E)**. CellChat-based results show the association of OPC cell populations with other cell populations in the collagen signaling pathway network.

Since we obtained different cell types in the mouse cerebrovascular tissue, we used non-negative matrix factorization (NMF) to explore crucial genes of the cellular transcriptional program (see Methods). A total of 16 components were selected in the NMF (Supplementary Figure S3A). After filtering the spectra at 0.2, 14/160 (9%) of crucial genes were removed before clustering (Supplementary Figure S3B). The heat map showed that the cells clustered well after NMF analysis (Supplementary Figure S3C). Next, NMI was used to examine the heterogeneity in the single-cell profiles of the mouse cerebral vasculature (Marjanovic et al., 2020). We found significant differences in AC, CPC, EC, EPC, MG and glial cells of the CNS between the older and younger groups according to the analysis of transcriptional heterogeneity (Figure 3A). CPC showed the most significant heterogeneity between the older and younger groups. We performed gene differential analysis at the single cell level to calculate DEGs between the older and younger groups (Figure 3B).

These genes are enriched in the KEGG pathways associated with Parkinson’s disease, phagosome, and *tuberculosis* in the young CPC group (Figure 3C). These genes were also enriched in GO-functional MF (ribonucleoprotein complex binding, MHC class I protein binding, and cell adhesion molecule binding), CC (endocytic vesicle, phagocytic vesicle, and ficolin-1-rich granule), and BP (neutrophil degranulation, neutrophil-mediated immunity and neutrophil activation involved in immune response) (Figure 3C). The genes upregulated in the young CPC group were enriched in the human T-cell leukemia virus 1 infection, osteoclast differentiation, and ribosome KEGG pathways (Figure 3D). These genes are also enriched in GO-functional MF (RAGE receptor binding, mRNA 5′-UTR binding, and structural constituent of ribosome), CC (cytosolic part, ribosomal subunit, and cytosolic ribosome), and BP (cotranslational protein targeting to membrane, nuclear-transcribed mRNA catabolic process nonsense-mediated decay, and SRP-dependent cotranslational protein targeting to membrane) (Figure 3D). CellChat was used to identify the primary senders, receivers, mediators, and influencers in the intercellular communication network, including secreted signaling, ECM receptors, and cell-cell contact (Supplementary Figure S4A–C). We found that OLG plays the role of an influencer and mediator in the collagen signaling pathway network, while AC, EPC, MAC, MNC, and SMC play the role of receiver in the collagen signaling pathway network, and finally, CPC functions as an influencer (Figure 3E and Supplementary Figure S4B). Through cellular interactions, CPCs are involved in altering cerebrovascular cells’ biological functions during the aging process. The heterogeneity of CPC, a neuroglial cell, differed most significantly from one level of aging to another and was influenced and regulated by intercellular signaling.

### Differential metabolic pathways and key genes of CPC in the older and younger groups

**FIGURE 4:**
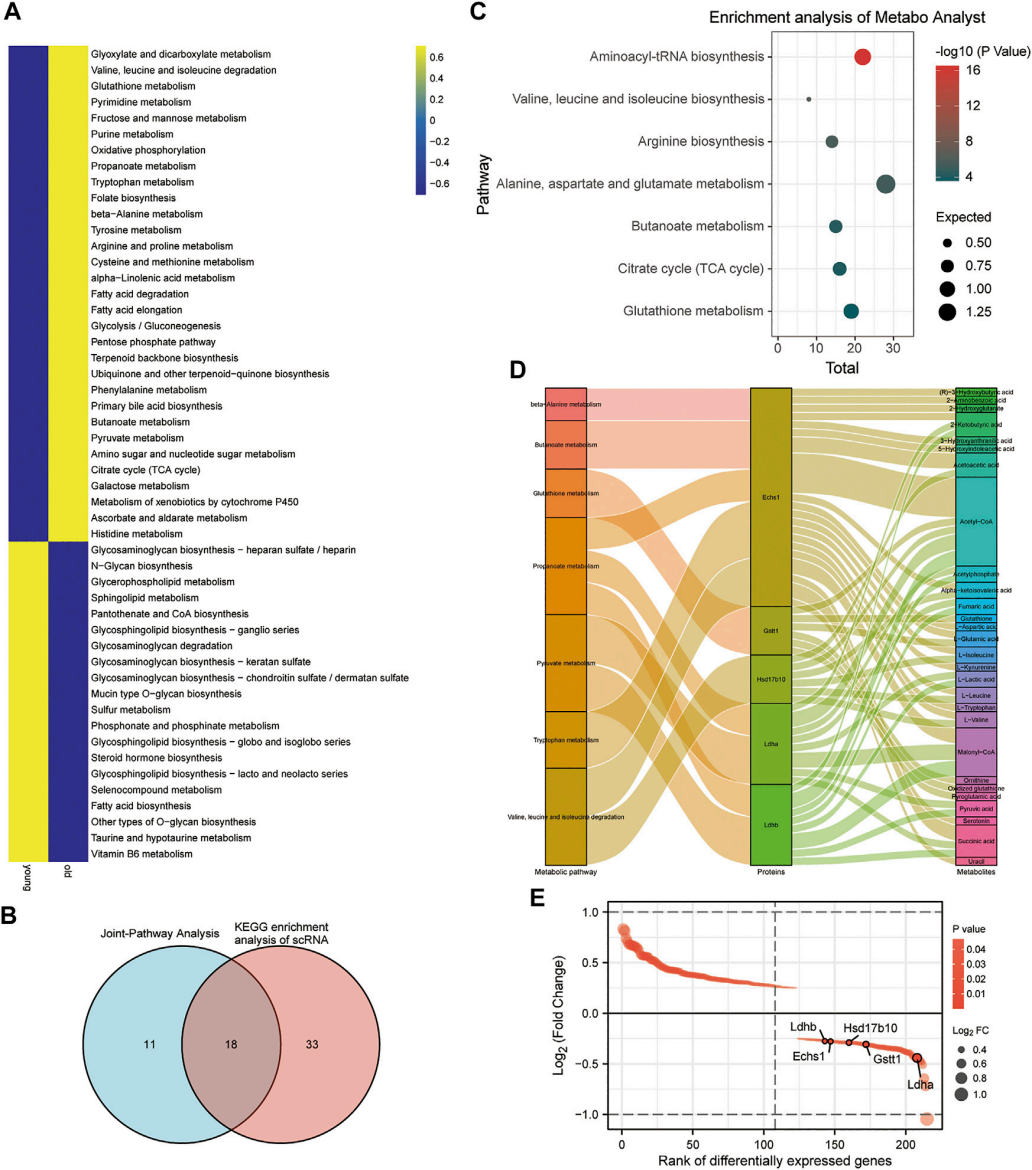
Metabolic differences in mouse brain CPC in the older versus younger groups. **(A)** Heat map showing CPC metabolic pathway scores based on scMetabolism. **(B)** Venn diagram showing 29 metabolic pathways in CPC (shown in Figure 1) enriched by MetaboAnalyst’s Joint-Pathway Analysis and 18 intersections of 51 metabolic pathways from KEGG based on scMetabolism. **(C)** Blister plots showing 7 of the 18 intersecting pathways (Figure 4B) are upregulated in the activity of aged mouse CPC. **(D)** Sankey plots showing the seven metabolic pathways regulated by five CPC age-related differential genes (*Ldhb, Echs1, Hsd17b10, Gstt1,* and *Ldha*) and the differential metabolites they affect. **(E)** Demonstrates the rank of the five age-related differential CPC genes (*Ldhb, Echs1, Hsd17b10, Gstt1,* and *Ldha*) downregulated in the younger group.

With scMetabolism, scRNA-seq metabolic analysis was performed to investigate the effects of aging on CPC metabolic pathways. Based on scMetabolism, 51 differential metabolic pathways were identified (Figure 4A). According to Koay YC et al., there were differences in circulating metabolites after exercise (Koay et al., 2021). MetaboAnalyst’s Joint-Pathway Analysis was used to enrich and reveal 29 metabolic pathways that exercise might affect in CPC. Based on Joint-Pathway analysis and KEGG enrichment analysis of scRNA, 18 metabolic pathways were obtained (Figure 4B). In the CPC of aged mice, seven of these metabolic pathways (including: Aminoacyl-tRNA biosynthesis; Valine, leucine, and isoleucine biosynthesis; Arginine biosynthesis; Alanine, aspartate and glutamate metabolism; Butanoate metabolism; Citrate cycle (TCA cycle); and Glutathione metabolism) were significantly upregulated (Figure 4C). The gene expression of these seven CPC metabolic pathways regulated by exercise was presented in a heat map (Supplementary Figure S5A–F). The multi-omics analysis revealed five CPC age-related differential genes (*Ldhb, Echs1, Hsd17b10, Gstt1*, and *Ldha*), and their differential metabolites regulated seven exercise-related metabolic pathways (Figure 4D). These differential genes were downregulated in the younger group and upregulated in the older group (Figure 4E). Therefore, Ldhb, Echs1, Hsd17b10, Gstt1 and Ldha may constitute key enzymes that alter CPC metabolism in old age and may be affected by exercise.

### Exercise metabolites target CPC differential proteases involved in cell differentiation

**FIGURE 5:**
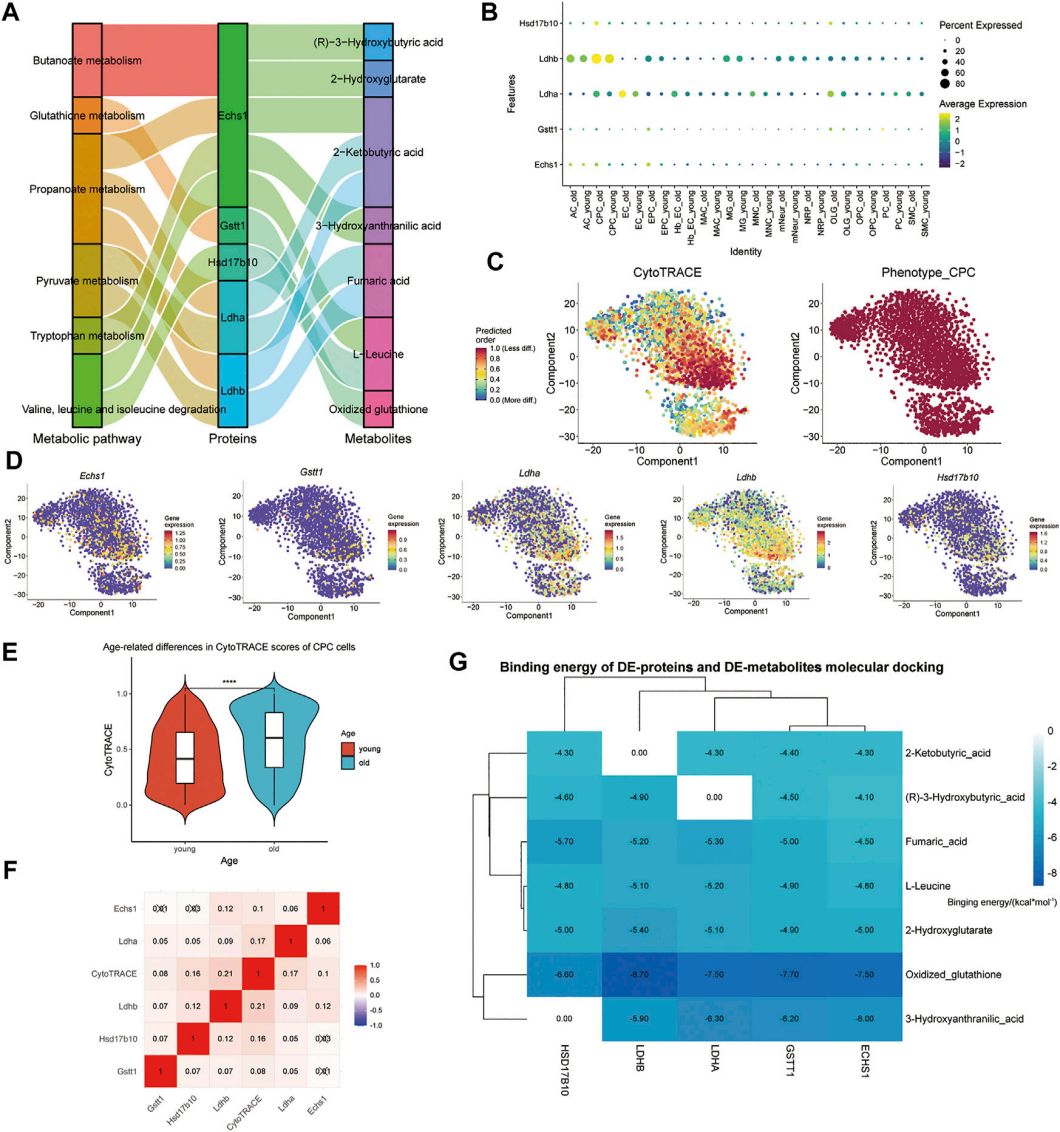
Exercise metabolites targeting aging-related metabolic differential proteases in mouse brain tissue CPC may affect the relative differentiation status of cells. **(A)** Sankey plots show the seven metabolic pathways regulated by the five CPC age-related differential genes (*Ldhb, Echs1, Hsd17b10, Gstt1,* and *Ldha*) and the associated metabolites upregulated by exercise. **(B)** Dot size indicates the percentage of cells expressing each gene, and dot color represents each group’s average expression level. The vertical axis lists CPC’s key metabolic enzyme genes, respectively. **(C)** CytoTRACE is used to show the relative differentiation status of CPC. **(D)** Expression of five CPC age-related differential genes (*Ldhb, Echs1, Hsd17b10, Gstt1,* and *Ldha*); **(E)** Cells of young and old CPC cells are shown in a violin plot according to their CytoTRACE scores; **(F)** Five genes with CPC age-related associations (*Ldhb, Echs1, Hsd17b10, Gstt1* and *Ldha*) are shown in the heatmap; **(G)** Heat map showing the binding free energy of DE-proteins and motility-associated DE-metabolites after molecular docking.

By binding to a target protein, small molecule compounds could inhibit its biological activity. Exercise may increase levels of some metabolites that target binding to key enzymes, which may prevent aging. Five CPC age-related differential proteins (Ldhb, Echs1, Hsd17b10, Gstt1, and Ldha) regulate these seven metabolic pathways, and their associated metabolites were upregulated by exercise (Figure 5A). The expression levels of selected marker genes were highly expressed in the older group and low in the younger group in CPC (Figure 5B). The higher the score in the CytoTRACE plot, the lower the differentiation status of the cells. We found that high expression of these five CPC age-related differential genes (*Ldhb, Echs1, Hsd17b10, Gstt1*, and *Ldha*) was associated with a lower differentiation status (Figure 5C,D). Exercise-related small molecule metabolites (including: (R)-3-Hydroxybutyric acid, 2-Hydroxyglutatate, 2-Ketobutyric acid, Fumaric acid, 3-Hydroxyanthranilic acid, and oxidized glutathione) are shown in Supplementary Figure S6. CytoTRACE scores were higher in older CPC cells, and five age-related genes (*Echs1*, *Hsd17b10*, *Gstt1, Ldha,* and *Ldhb*) correlated strongly with CytoTRACE scores (Figures 5E,F). This suggests that higher CytoTRACE scores are positively associated with aging and high expression of *Echs1*, *Hsd17b10*, *Gstt1, Ldha,* and *Ldhb*. The free energy of binding of the small metabolic molecule GSSG to those key enzymes were less than −6.5 kcal/mol (Figure 5G). Exercise increases GSH levels in the blood while the GSSG levels decrease. GSSG is a metabolite of glutathione (GSH). Thus, physical exercise might reduce CPC aging-related metabolism by reducing GSSG targeting to ECHS1, GSTT1, LDHA, and LDHB.

### MDS of GSSG targets ECHS1, GSTT1, LDHA and LDHB

**FIGURE 6:**
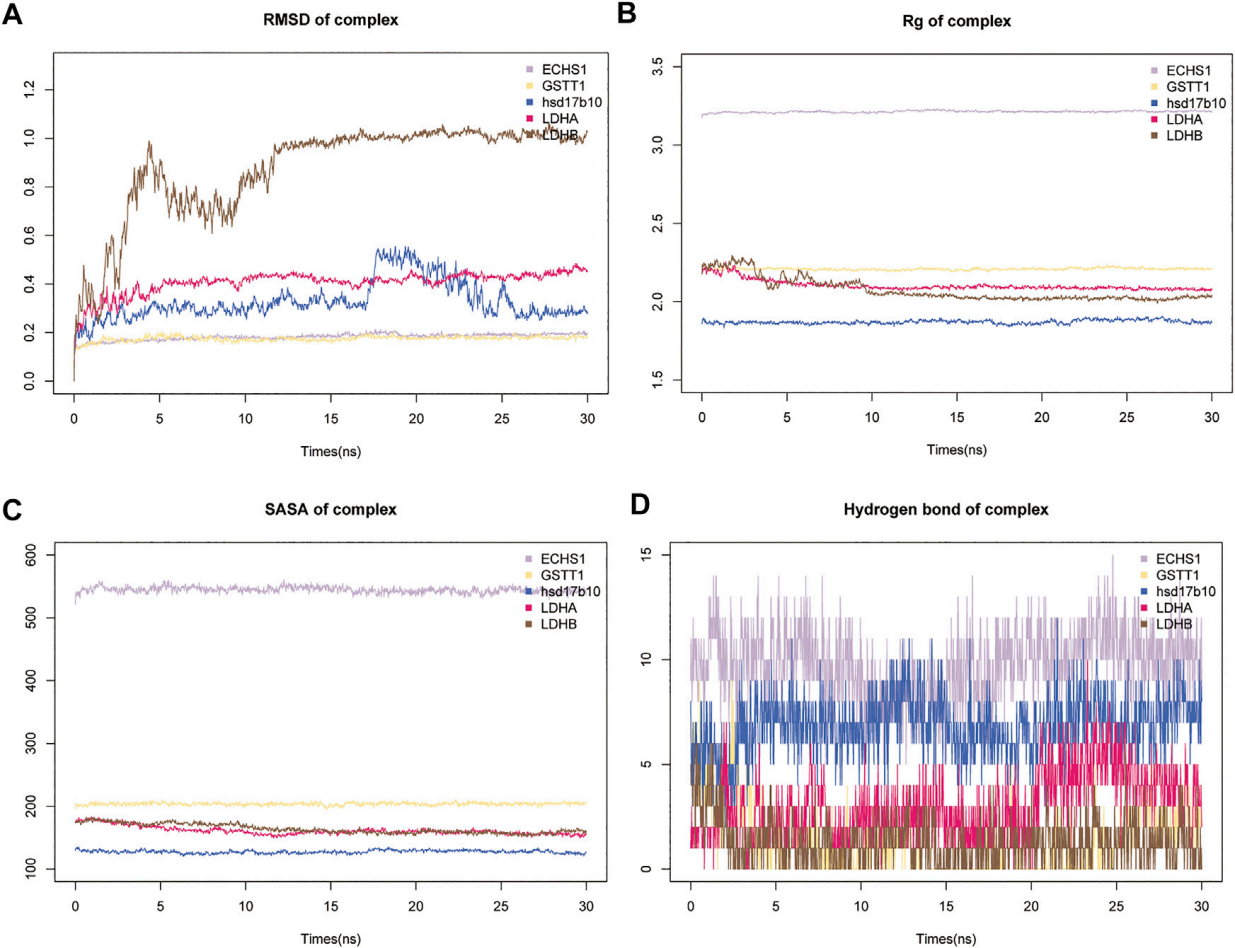
Results of molecular dynamics simulations (MDS) of five key metabolic enzymes. **(A)** The atomic root mean square deviation (RMSD) of the protein-metabolite complex MDS. **(B)** Rg variation of protein-metabolite complex MDS. **(C)** Solvent accessible surface area (SASA) variation of proteins in 0–3 ns of protein-metabolite complex MDS. **(D)** Changes in hydrogen bonding in the steady state of protein-metabolite complexes.

MDS is an important method for studying the stability and kinetics of complexes in aqueous solutions. Based on the RMSD values of MDS, all systems can be stabilized after MDS (Figure 6A). Rapid stabilization of ECHS1, GSTT1, and LDHA suggests that their docking results are more suitable, and they finally stabilize at 0.2, 0.2, and 0.4 nm (Figure 6A). After the fluctuation at 17 ns, the distance between LDHB and GSSG converged to 1 nm, and the distance between HSD17B10 and GSSG converged to 0.3 nm (Figure 6A). LDHB demonstrated only a slight fluctuation at 10 ns in the Rg analysis (Figure 6B). There are solvent-accessible surfaces on proteins, and the solvent accessible surface area (SASA) of the protein is very stable from 0 to 30 ns, indicating favorable binding and progressive protein tightening (Figure 6C). The variation of hydrogen bonding curves showed that the number of hydrogen bonds formed by binding to GSSG was ranked as ECHS1 > HSD17B10 > LDHA > GST1 > LDH (Figure 6D).

### GSSG-ECSH1 secondary structure analysis and MM/GBSA

**FIGURE 7:**
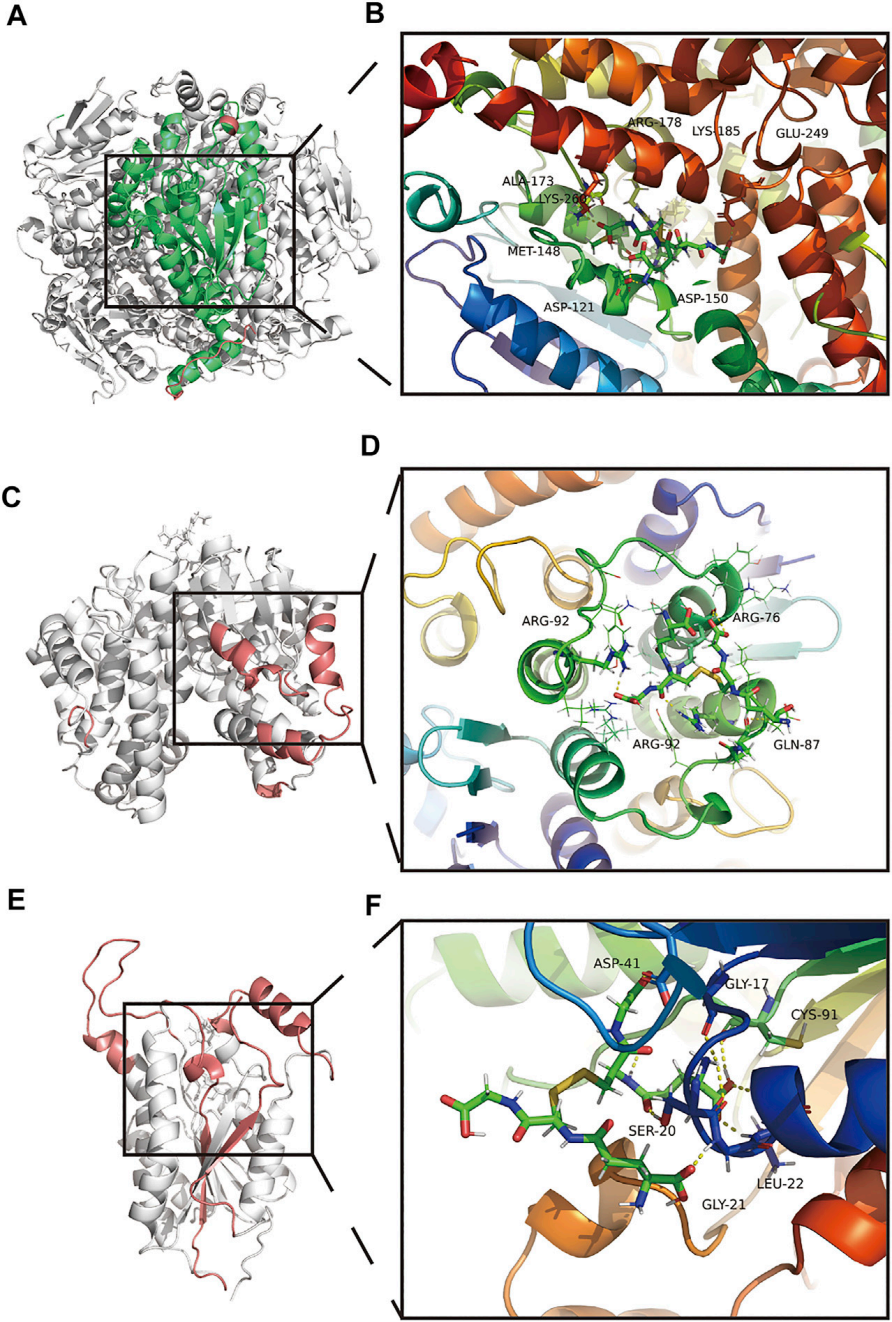
Amino acid interactions of the stable structure. **(A,B)** Amino acid interactions of GSSG-ECSH1 complex MDS after obtaining the stable structure. **(C,D)** Amino acid interactions of GSSG-GSTT1 complex MDS after obtaining the stable structure. **(E,F)** Amino acid interactions of GSSG-HSD17B10 complex MDS after obtaining the stable structure.

After MDS, the secondary structure of the GSSG-ECSH1 complex changed, with a greater amount of turn, bend, A-helix, and lesser number of 3-helix (Supplementary Figure S7A). GSSG-ECSH1 fluctuation sites are 31–32, 101–102, 281–290 and eight amino acids were involved in the interaction of the GSSG-ECSH1 complex, namely ASP121, MET-148, ASO-150, ALA-173, ARG-178, LYS-185, GLU-249, and LYS-266 (Figure 7A,B). ECSH1 and GSSG are likely to interact in the GSSG-ECSH1 complex (Supplementary Figure S7B). When each contact residue of the GSSG-ECSH1 complex was broken down, we found THR-124, MET-148, ASP-150, and LYS-121 of the B-chain to facilitate binding, while ARG-178, LYS-241, and CYS-149 of the B-chain hindered binding (Supplementary Figure S7C). In the stable structure of GSSG-ECSH1, MET-148 and ARG-178 are directly involved in the interaction between ECSH1 and GSSG. Therefore, the above analysis suggests that GSSG can stably bind ECSH1.

### GSSG-GSTT1 secondary structure analysis and MM/GBSA

After MDS, the secondary structure of the GSSG-GSTT1 complex decreased; coils, bends, 5-helix increased, and turn, A-helix decreased (Supplementary Figure S7D). GSSG-GSTT1 fluctuated at sites 35–47, 128–131, 209–230, 183–186 and amino acids involved in the interaction of the GSSG-GSTT1 complex were ARG-76 (A-chain), GLN-87 (A-chain), ARG-92 (A-chain), and ARG-92 (B-chain) (Figure 7C,D). GSSG and GSTT1 had a negative total free energy, suggesting an interaction between GSTT1 and GSSG (Supplementary Figure S7E). ARG-92 and GLN-87 of the B chain hindered binding of the GSSG-GSTT1 complex, while ASP-88 and LEU-89 facilitated binding (Supplementary Figure S7F). The stability of the GSSG-GSTT1 complex was dependent on ARG-92 of the B chain, which interacts directly with GSTT1 and GSSG. These data suggest that GSSG could stably target and bind GSTT1.

### GSSG-HSD17B10 secondary structure analysis and MM/GBSA

GSSG-HSD17B10 complex after MDS showed that the overall structure increased, B-sheet, bend, 3-helix increased, and coil, 5-helix decreased (Supplementary Figure S7G). GSSG-HSD17B10 complex contained highly flexible sites 96–116, 143–163, 207–223, 245–261, and GSSG-HSD17B10 had five amino acid interactions, namely GLY-17, SER-20, GLY-21, LEU-22, ASP-41, and CYS-91 (Figures 7E,F). HSD17B10 and GSSG are likely to interact, and VDWAALS and EEL suggest that both water and electrostatic interactions contribute to binding, while ESURF and EGB suggest that polar solubilization does not contribute to binding (Figure 7H). The binding ability between GSSG and HSD17B10 gradually decreased with each contact residue; ASP-41 and LEU-42 of the A-chain played a hindering role (Supplementary Figure S7I). In the stable structure of the GSSG-HSD17B10 complex, GLY-21, LEU-22, and ASP-41 were directly involved in the interaction between HSD17B10 and GSSG. These results suggested a stable interaction between HSD17B10 and GSSG.

### GSSG-LDHA secondary structure analysis and MM/GBSA

**FIGURE 8:**
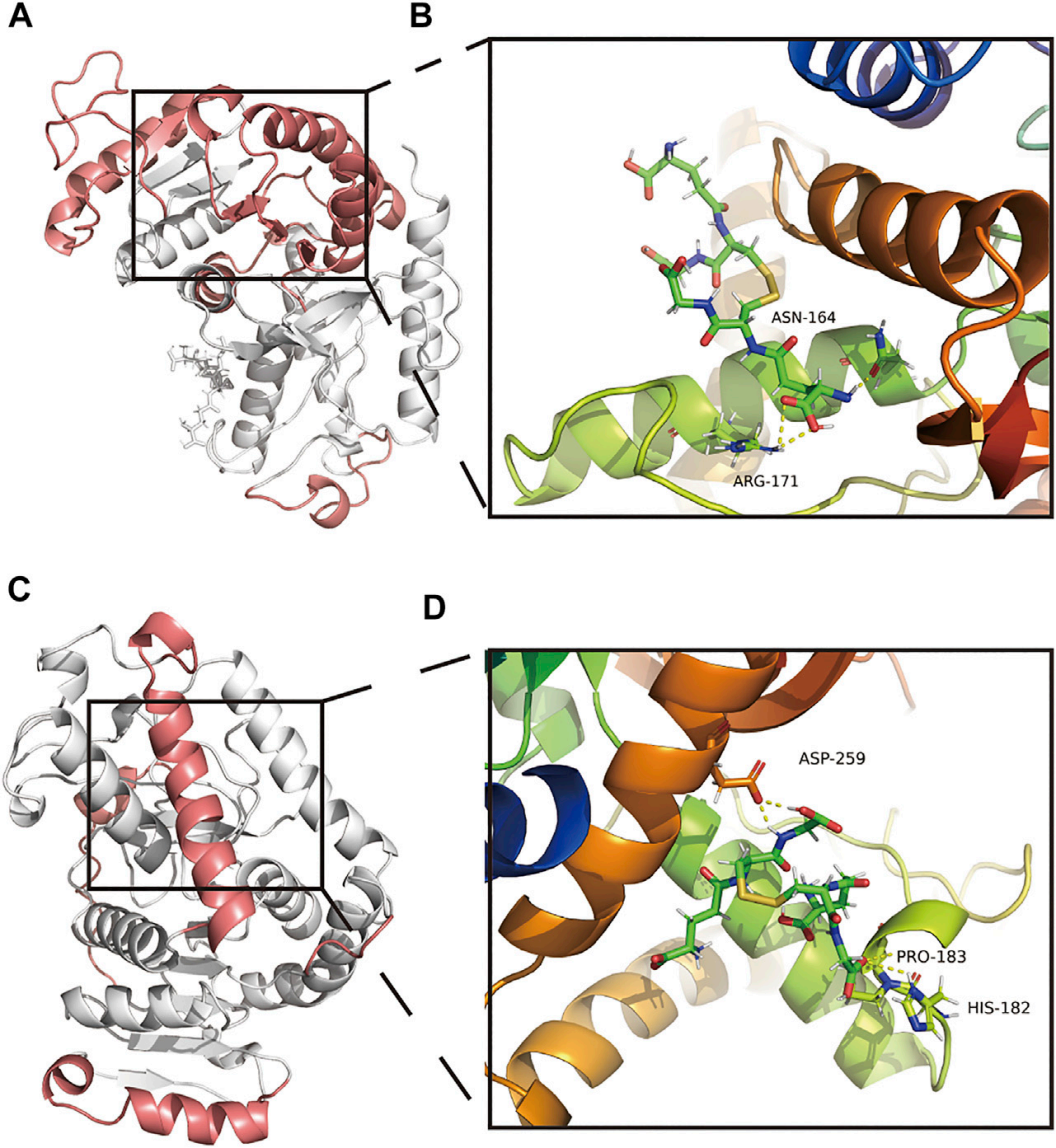
Amino acid interactions of the stable structure. **(A,B)** Amino acid interactions of GSSG-LDHA complex MDS after obtaining stable structures. **(C,D)** Amino acid interactions of GSSG-LDHB complex MDS after obtaining stable structures.

The secondary structure of the GSSG-LDHA complex after MDS could be seen as decreased overall structure, increased B-sheet, bend, and 3-helix, and decreased A-helix (Supplementary Figure S8A). There were five highly flexible sites in the GSSG-LDHA complex: 1–20, 54–163, 207–223, 245–261, and two amino acids were involved in the GSSG-LDHA complex, including ANS164 and ARG-171 (Figures 8A,B). GSSG-LDHA complex had a negative total free energy, indicating that LDHA and GSSG might interact. VDWAALS and EEL indicated that water and electrostatic interactions contributed to the binding. In contrast, ESURF and EGB suggest that polar solubilization does not promote binding (Supplementary Figure S8B). In the GSSG-LDHA complex, ALA-168, LEU-183, SER-255, ARG-171, ARG-269, ARG-270, and LEU-254 of the A-chain were the primary contact residues that facilitate binding, while ARG-171, ARG-269, ARG-270, and LEU-254 acted as barriers (Supplementary Figure S8C). Among them, ARG-171 directly interacted with LDHA and GSSG in the stable structure of GSSG-LDHA. Therefore, these results suggest that GSSG is capable of targeting LDHA stably.

### GSSG-LDHB secondary structure analysis and MM/GBSA

According to the secondary structure of the GSSG-LDHB complex after MDS, the overall structure decreased, coils, bends, and turns increased, and B-sheets decreased (Supplementary Figure S8D). GSSG-LDHB complexes had highly flexible sites 1–20, 55–73, 100–104, 221–249, three amino acid interactions existed in the GSSG-LDHB complex, including HIS-182, PRO-182, and ASP-259 (Figures 8C,D). The GSSG-LDHA complex had a negative total free energy, suggesting that LDHA and GSSG are likely to interact. VDWAALS and EEL suggested water and electrostatic interactions contribute to binding, while ESURF and EGB suggested polar solubilization was not conducive to binding (Supplementary Figure S8E). ARG-172, ARG-270, ARG-270, and ASN-165 of the A-chain mainly hindered binding, while SER-256, ALA-252, LEU-166, and ASP-256 of the A-chain were favorable contacts (Supplementary Figure S8F). In the stable structure of the GSSG-LDHB complex, ASP-256 was directly involved in the interaction between LDHB and GSSG. However, according to the animation of MDS, GSSG-LDHB complex does not seem to bind very well. Despite their potential interactions, GSSG-LDH may not be very stable complexes.

## Discussion

Exercise has been identified as an effective method for preventing aging-related neurodegeneration. In the present study, we found that exercise improved the metabolic landscape of the cerebrovascular microenvironment. We found that ECHS1, GSTT1, HSD17B10, LDHA, and LDHB were proteins associated with CPC age heterogeneity and involved in the metabolism of (R)-3-Hydroxybutyric acid, 2-Hydroxyglutarate, 2-Ketobutyric acid, 3-Hydroxyanthranilic acid, Fumaric acid, L-Leucine, and Oxidized glutathione. MDS simulations suggested that GSSG might cause metabolic abnormalities in NVU due to CPC age heterogeneity.

Brain cells vary in type and regulation during aging, but little is known about how they change. Research has shown that energy depletion during brain aging is associated with heterogeneous cellular regulatory networks (Davie et al., 2018). This study refines these studies and reveals heterogeneity in NMI scores and transcriptomes during brain aging. These age heterogeneity-associated neurodegenerative disorders are closely associated with neuropathologies such as glucose metabolism disorders in the whole brain or specific cells (Dringen, 2000). According to recent findings, glutathione metabolism and reactive oxygen species defense in glial cells play an important role (Dringen, 2000). While glutathione plays an important role in redox signaling, the role of GSSG has not been completely established (Mailloux and Treberg, 2016; Méndez et al., 2016; Zhang et al., 2012; Flohé, 2013). GSSG occurs when reactive oxygen species react with glutathione (GSH), a natural antioxidant that prevents oxidative damage in the body (Yu et al., 2012; Cramer, 2021). Moreover, GSSG/GSH can be used as an analytical tool to reveal redox metabolic disorders caused by glutathione (Masters et al., 2020; Neese et al., 2020). In neuropsychiatric and neurodegenerative diseases, GSH plays an important role (Gu et al., 2015). GSSG/GSH levels are higher in the whole blood of AD patients because their cellular redox status has been altered (Martínez de Toda et al., 2019). Muscular dystrophy and metabolic syndrome are also associated with dysregulation of GSH/GSSG balance (Pérez-Torres et al., 2017). Studies have focused on the redox function of GSH, while an understanding of the biochemical functions of GSSG has been limited. First, we investigated whether elevated GSSG in the ADRD microenvironment directly targets CPC age heterogeneity-related proteins (ECHS1, GSTT1, HSD17B10, LDHA, and LDHB), suggesting that brain energy metabolism may be diminished. The sections below discuss how ECHS1, GSTT1, HSD17B10, LDHA, and LDHB function in the brain and vascular health.

Short-chain enoyl-CoA hydratase (ECHS1) is a mitochondrial matrix enzyme that plays several roles, such as oxidizing fats and metabolizing essential amino acids, including valine (Haack et al., 2015). ECHS1 deficiency, for instance, can cause secondary pyruvate dehydrogenase deficiency, resulting in clinical symptoms (Ferdinandusse et al., 2015). ECHS1 deficiency leads to mitochondrial encephalopathy, as shown by delayed motor and cognitive development and abnormal brain MRI signals in the nucleus accumbens and caudate nucleus (Huffnagel et al., 2017). ECHS1 deficiency also causes paroxysmal exercise-induced dyskinesias (PED) (Mahajan et al., 2017). Therefore, ECHS1 expression in the CPC of aged rats might serve as a protector against injury. Furthermore, exercising may protect the function of ECHS1 and thus improve NVU energy metabolism by reducing GSSG levels in circulating blood.

We found that the oxidative stress-related factors, such as aging, upregulate the Glutathione S-transferase theta 1 (GSTT1) level in human cells (Ito et al., 2011). GSTT1 functions to bind electrophile compounds to glutathione, allowing them to proceed to the next step in metabolism. Examples include drugs, environmental toxins, and oxidative chain products. GSTT1 functions as an anticancer agent in the body by cleaning up environmental toxins and carcinogens (Geng et al., 2016). Researchers found that avoiding nitrous compounds during pregnancy and abstaining from GSTT1 consumption may reduce children’s risk of brain tumors (Nielsen et al., 2011). Multiple sclerosis, refractory schizophrenia, and osteosarcoma have all been linked to polymorphisms in GSTT1 (Pérez-Torres et al., 2017; Martínez de Toda et al., 2019; Pinheiro et al., 2017; Wang et al., 2015). Molecular dynamics studies revealed that increased levels of GSSG might inhibit the activity of GSTT1, impairing its function and affecting GSTT1 metabolism in NVU.

17β-Hydroxysteroid dehydrogenase type 10 (HSD10), encoded by the *HSD17B10* gene at Xp11.2, is a mitochondrial NAD + -dependent dehydrogenase that catalyzes multiple reactions and binds to many proteins and peptides (Yang et al., 2011). Missense mutations resulting in 17β-HSD10 deficiency cause infantile neurodegeneration characterized by progressive psychomotor disability and altered mitochondrial morphology (Yang et al., 2014). Generally, ADRD patients have abnormally elevated levels of 17-HSD10, and steroid endostasis can be restored by using neuroactive steroids or by modulating 17-HSD10 activity to protect neurons (Ferdinandusse et al., 2015; Huffnagel et al., 2017; He et al., 2019). This study shows that GSSG can impede the function of key enzymes in the NVU of ADRD patients, and that prolonged physical exercise can reduce GSSG levels, reverse the negative effects of GSSG, and improve NVU metabolism.

Lactate dehydrogenase, a NAD-dependent kinase, contains three subunits, LDHA, LDHB, and LDHC, which can form six tetrameric isozymes that catalyze the oxidation of lactate to pyruvate (Urbańska and Orzechowski, 2019). LDHA preferentially converts pyruvate to lactate under anaerobic conditions, while LDHB preferentially converts lactate to pyruvate when oxygen is present (Urbańska and Orzechowski, 2019). As metabolic links between tumor and stroma, LDHA and LDHB play essential roles in tumor cell metabolism and adaptation to unfavorable environments, as well as regulating cell death (Massari et al., 2016; Mishra and Banerjee, 2019; Urbańska and Orzechowski, 2019). LDHA and LDHB levels may increase when brain lactate levels and lactate transport decrease. Although these compounds are meant to ameliorate deficits in brain energy metabolism, they might block lactate trafficking from glial cells (Zhang et al., 2018). Physical exercise might improve this state by reducing the GSSG/GSH ratio by targeting LDHA in GSSG-targeted therapies.

Neurodegeneration associated with aging can be prevented through physical activity. We measured the heterogeneity between cells using NMF and NMI methods. We also identified age-related metabolic pathways in CPC through intercellular interactions and combined multi-omics analysis. Our results suggest that GSSG might directly target CPC age heterogeneity-related proteins (ECHS1, GSTT1, HSD17B10, LDHA, and LDHB) by molecular docking and MDS. Accordingly, circulating GSSG might be involved in aging-related neurodegeneration in NVU, and this may be due to its ability to target age heterogeneity-associated proteins in CPC. In addition, future studies and more MDS simulation will need to investigate the molecular mechanism of action of GSSG *in vivo* and *in vitro*. Furthermore, future studies should examine the role of ESHS1, GSTT1, HSD17B10, LDHA, and LDHB in aging CPC expression. And more research is needed to learn how physical exercise affects CPC interaction with other cells, such as astrocytes, to improve NVU function. This research aids in pharmacological studies and drug development targeting neurodegenerative diseases.

## Conclusion

According to the present study, CPC metabolic pathway activity was changed in the elderly group. GSH metabolite and GSSG inhibit intracerebral energy metabolism in CPC by targeting age heterogeneity-related proteins (ECHS1, GSTT1, HSD17B10, LDHA, and LDHB), while exercise enhances it by improving the GSH/GSSG balance. Based on single-cell integration analysis, our molecular dynamics and free energy simulations confirmed that GSSG interferes with intracerebral energy metabolism.

## Data Availability

The datasets presented in this study can be found in online repositories. The names of the repository/repositories and accession number(s) can be found in the article/Supplementary Material.

## Supplementary material

Supplementary Figure S1 Quality control for the GSE147693 scRNA-seq dataset. **(A)** Differences in expression of signature RNA, the total number of RNAs detected, percent of mitochondrial counts (pMT), and percent of hemoglobin RNA counts (pHB) in different clinical samples. **(B)** Scatter plots showing the correlation between the number of RNAs detected and the number of mitochondria in a single cell (Left panel), the number of signature genes (middle panel), and the number of erythrocyte proteins mixed in the sample (right panel), respectively.

Supplementary Figure S2 The top 10 marker genes for each cell cluster are shown in a heat map. The marker genes of various cells are shown here.

Supplementary Figure S3 Analysis of cNMFs and selection of hyperparameters. **(A)** Diagnostic plots are used to determine the components that should be selected. **(B)** The cell score distribution was plotted with the Usage matrix to remove components with extreme distributions. **(C)** Heat map showing the clustering effect after calculating the cosine distance on NMF.

Supplementary Figure S4 Using CellChat to identify key senders, receivers, mediators, and influencers in intercellular communication networks. **(A)** secreted signaling-related CellChat functions, **(B)** ECM receptor-related CellChat functions, and **(C)** cell-cell contact-related CellChat functions.

Supplementary Figure S5 Heatmap of CPC metabolic pathway-related gene expression. **(A)** Heatmap of selected genes in the butanoate metabolism pathway; **(B)** Heatmap of selected genes in glutathione metabolism pathway; **(C)** Heatmap of selected genes in the propanoate metabolism pathway; **(D)**. Heatmap of selected genes in the pyruvate metabolism pathway gene heatmap; **(E)**. Heatmap of selected genes in the tryptophan metabolism pathway; **(F)**. Heatmap of selected genes in the leucine and isoleucine degradation pathway.

Supplementary Figure S6 The structures of six exercise-related small molecule metabolites [including: (R)-3-Hydroxybutyric acid, 2-Hydroxyglutatate, 2-Ketobutyric acid, Fumaric acid, 3-Hydroxyanthranilic acid, and oxidized glutathione].

Supplementary Figure S7 MDS secondary structure and binding free energy analysis. **(A)** Plots of individual secondary structures of proteins in the MDS system with time. **(B)** Binding free energy between GSSG-ECSH1 complexes. **(C)** Relationship between each contact residue and binding in the GSSG-ECSH1 complex. **(D)** Plot showing each secondary structure of the GSSG-GSTT1 complex with time in the MDS system. **(E)** Binding free energy between GSSG-GSTT1 complexes. **(F)** Relationship between each contact residue and binding in the GSSG-GSTT1 complex. **(G)** Plot showing the individual secondary structures of the GSSG-HSD17B10 complex in the MDS system with time. **(H)** The binding free energy between GSSG-HSD17B10 complexes. **(I)** The relationship between each contact residue and binding in the GSSG-HSD17B10 complex. Notes: Negative energy indicates strong binding ability. ‘Total’ indicates the system’s total energy, which represents the ability of the protein and small molecule to interact with each other. VDWAALS- van der Waals energy; EEl- Electrostatic energy; EGB- Polar solvation energy; ESURF- Non-polar solvation energy; GGAS- Total gas phase free energy; GSOLV- Total solvation free energy; TOTAL free energy- GSOLV + GGAS.

Supplementary Figure S8 MDS secondary structure analysis and binding free energy analysis. **(A)** Plots of the individual secondary structures of the GSSG-LDHA complex proteins in the MDS system over time. **(B)** Binding free energy between GSSG-LDHA complexes. **(C)** Relationship between each contact residue and binding in the GSSG-LDHA complex. **(D)** Plot illustrating the change in each secondary structure between GSSG-LDHB complexes with time in the MDS system. **(E)** Binding free energy between GSSG-LDHB complexes. **(F)** Relationship between each contact residue and binding in the GSSG-LDHB complex. Notes: Negative energy indicates a strong binding ability. “Total” indicates the system’s total energy and represents the ability of the protein and small molecule to interact. VDWAALS- van der Waals energy; EEl- Electrostatic energy; EGB- Polar solvation energy; ESURF- Non-polar solvation energy; GGAS- Total gas phase free energy; GSOLV- Total solvation free energy; TOTAL free energy- GSOLV + GGAS.
