# Supplementary figures and images for: Exercise improves choroid plexus epithelial cells metabolism to prevent glial cell-associated neurodegeneration
Source: Front Pharmacol. 2022 Sep 16;13:1010785. doi: 10.3389/fphar.2022.1010785 (PMC9523215; doi:10.3389/fphar.2022.1010785)

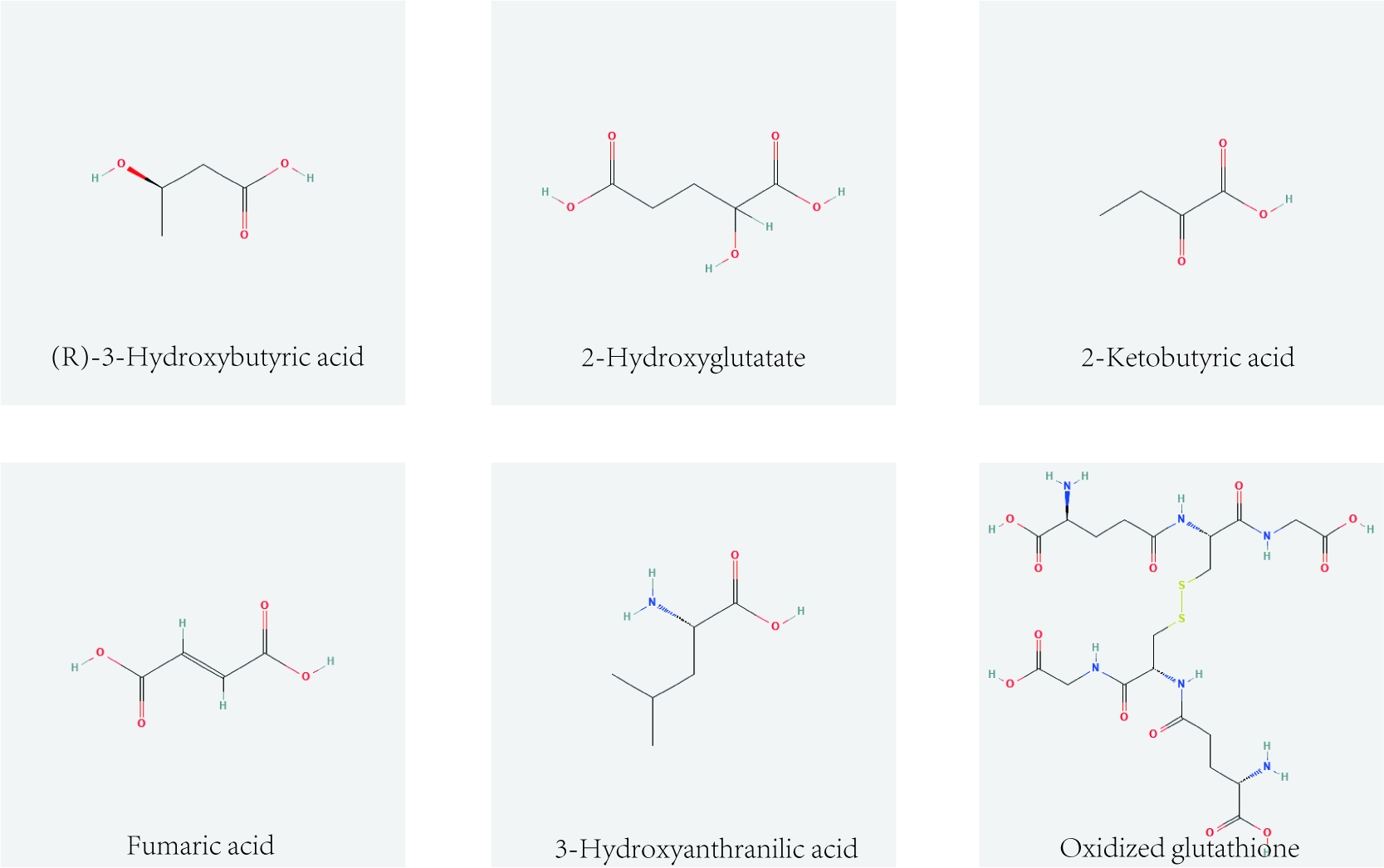

Supplement: Supplementary file 1 [file Image6.TIF]

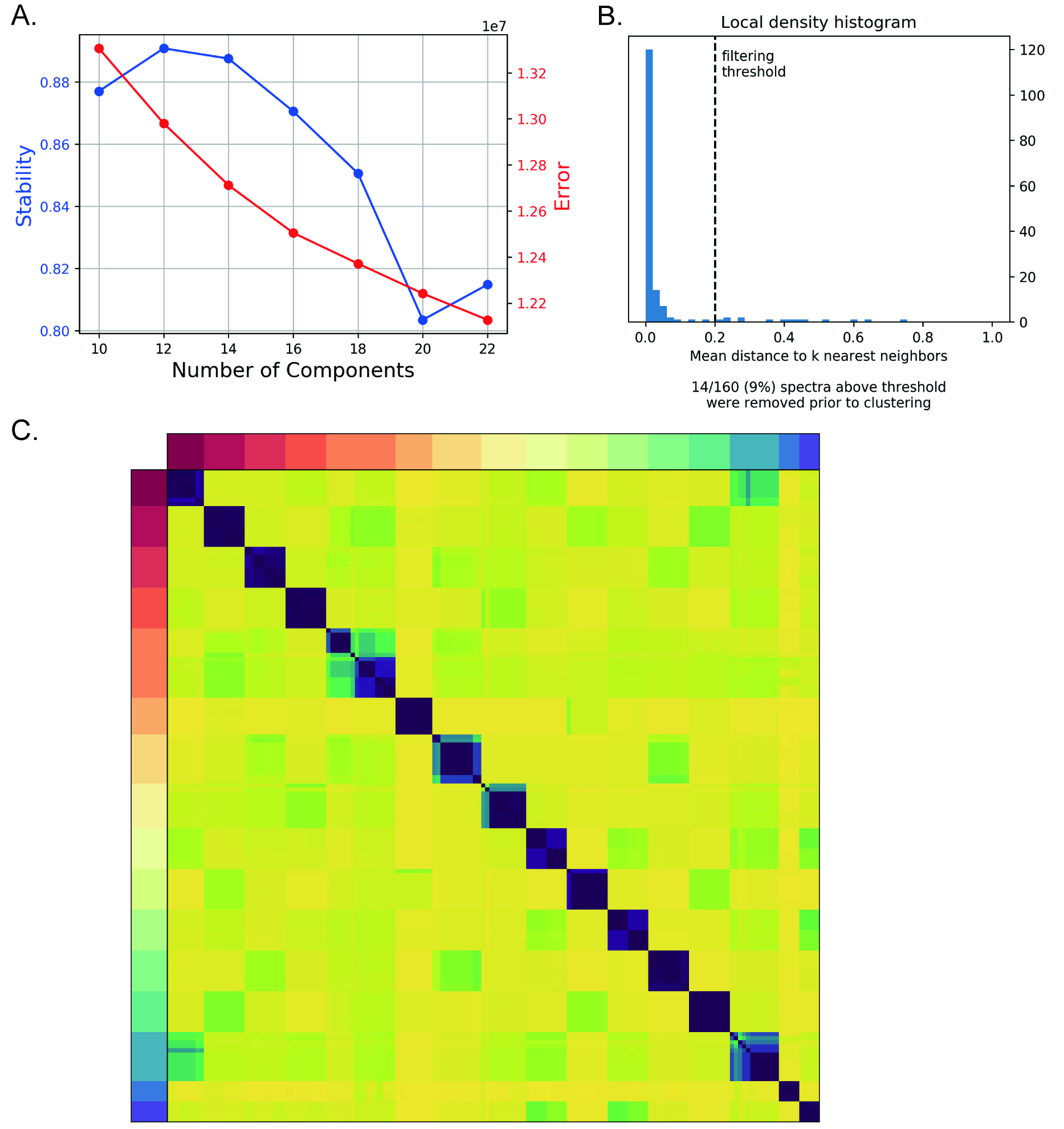

Supplement: Supplementary file 3 [file Image3.TIF]

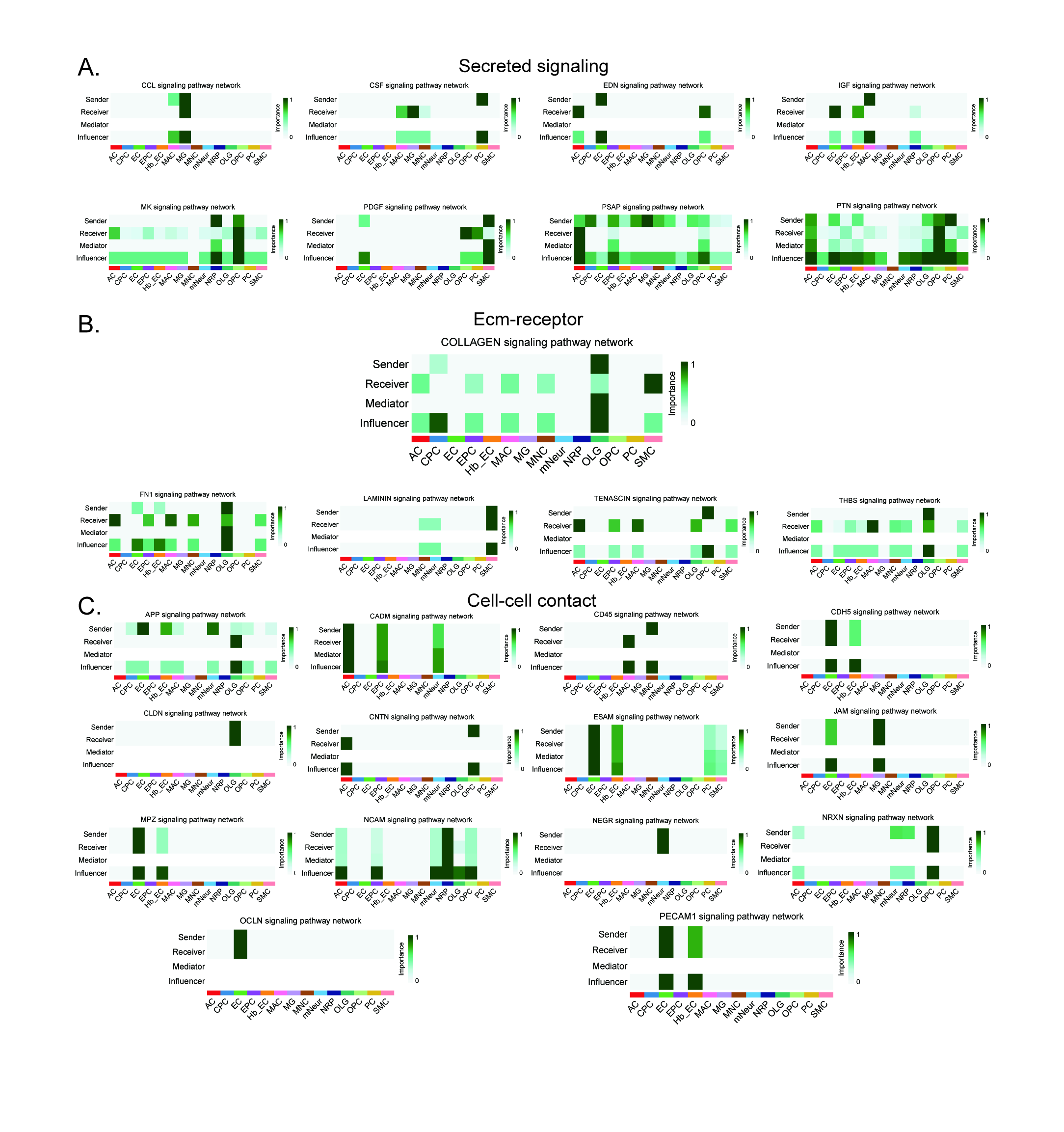

Supplement: Supplementary file 4 [file Image4.TIF]

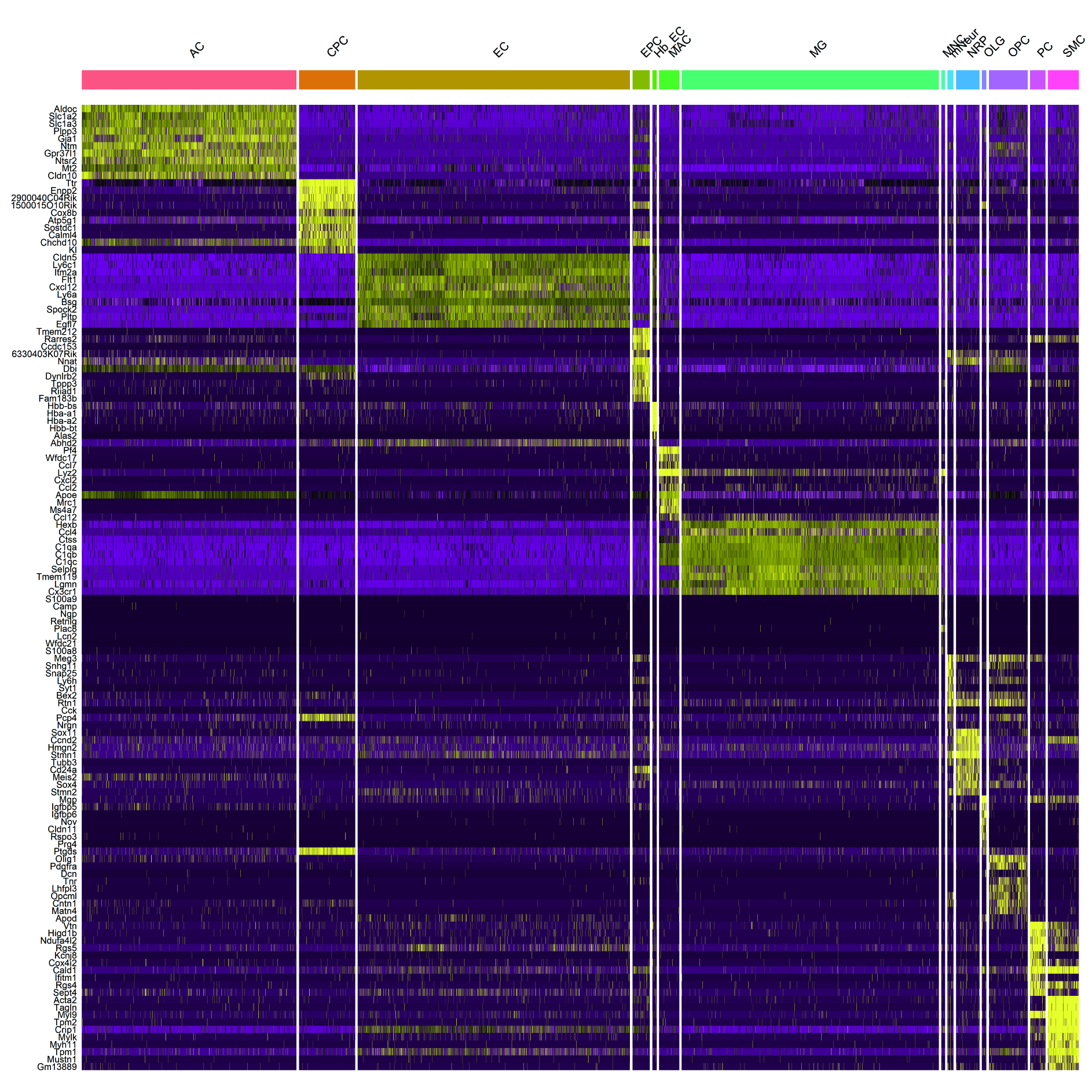

Supplement: Supplementary file 5 [file Image2.TIF]

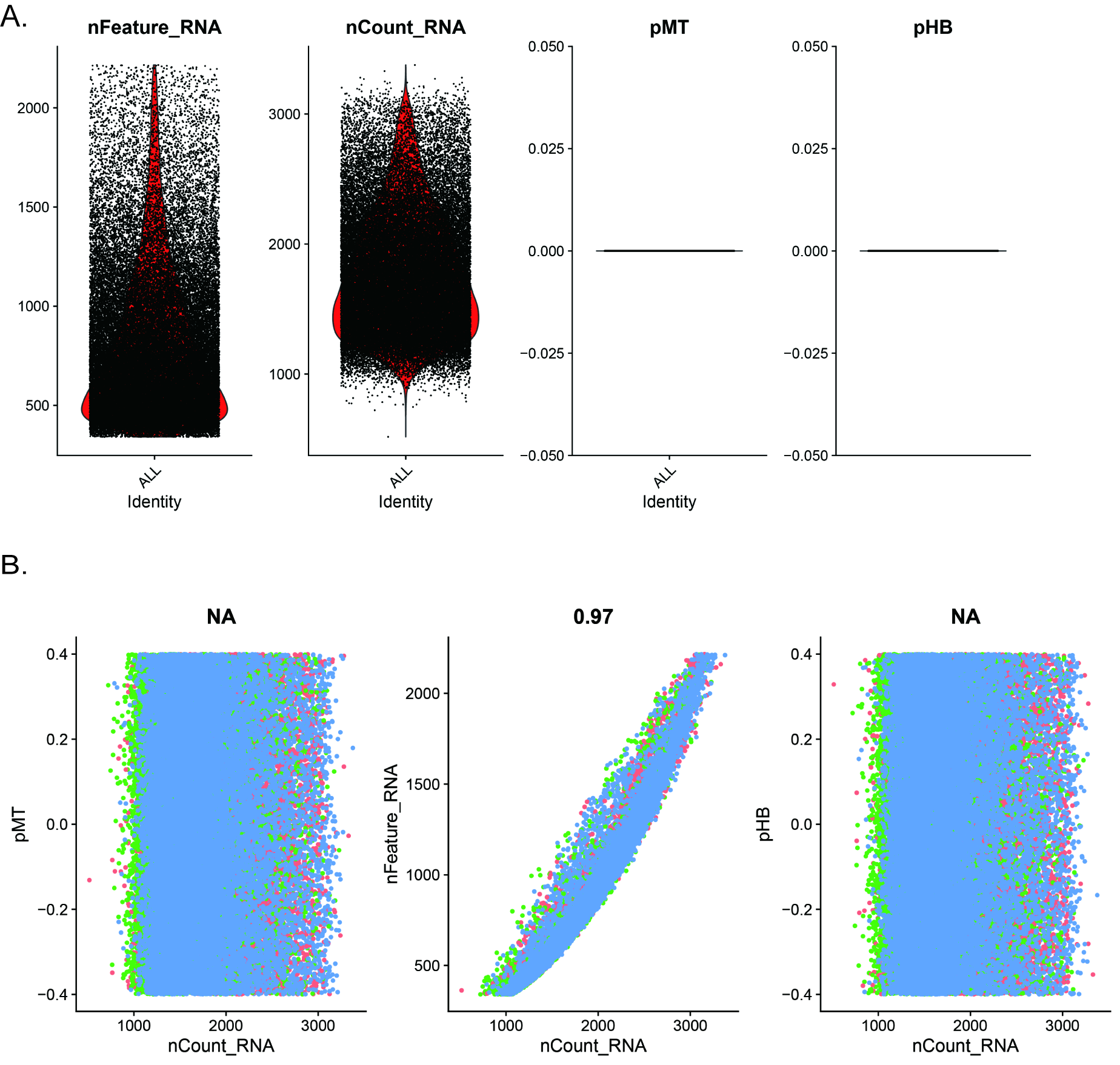

Supplement: Supplementary file 6 [file Image1.TIF]

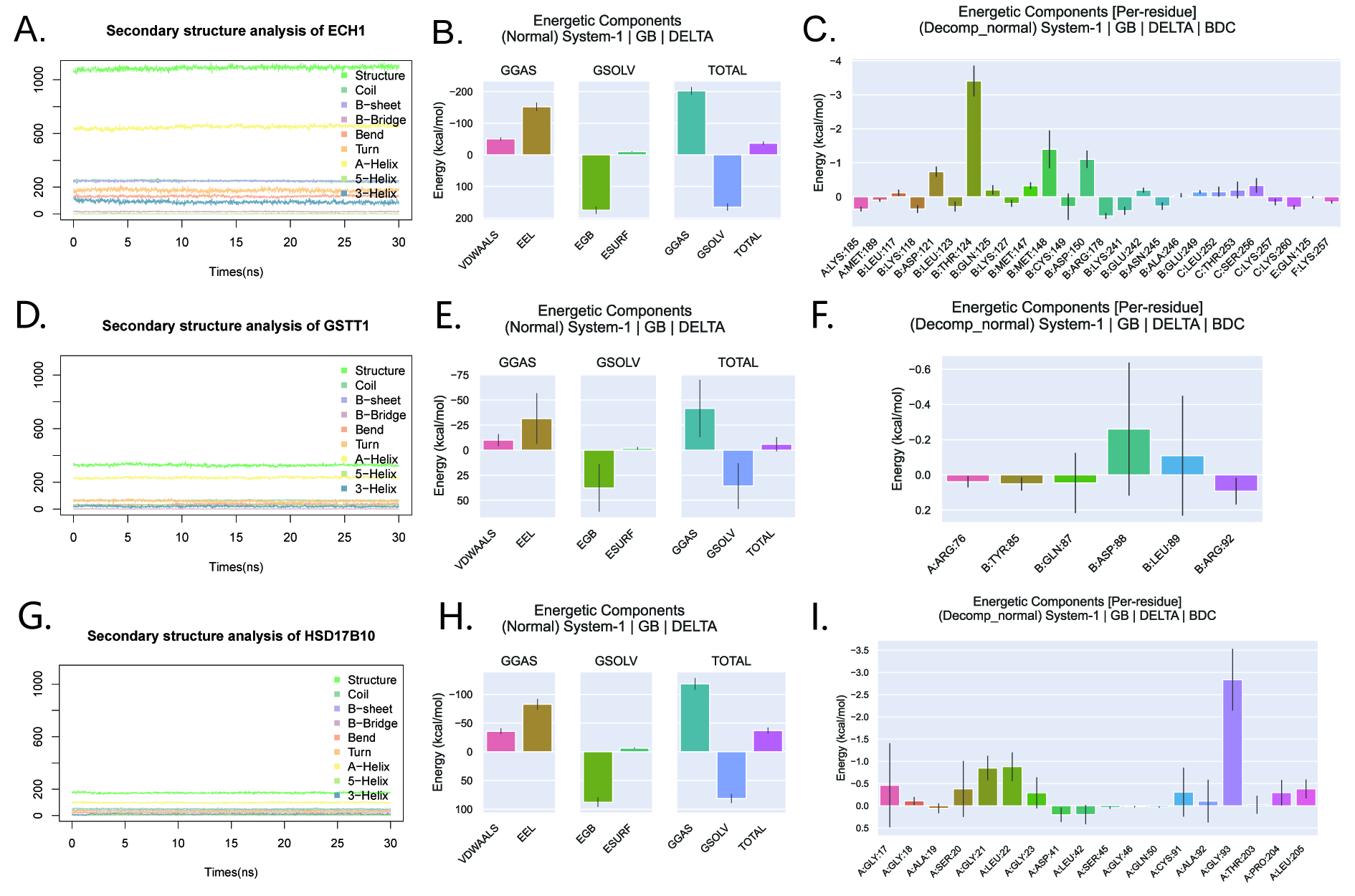

Supplement: Supplementary file 7 [file Image7.TIF]

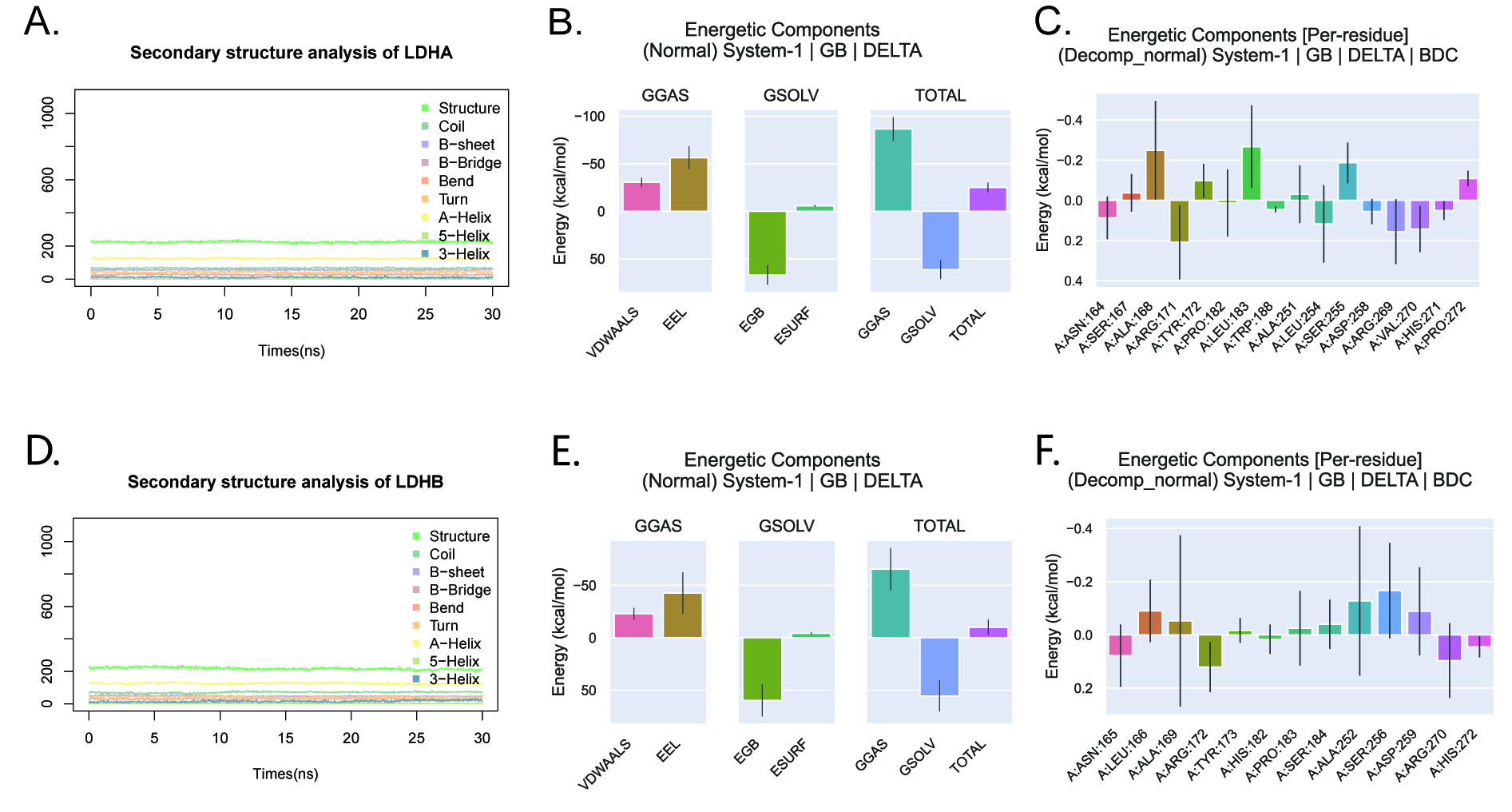

Supplement: Supplementary file 10 [file Image8.TIF]

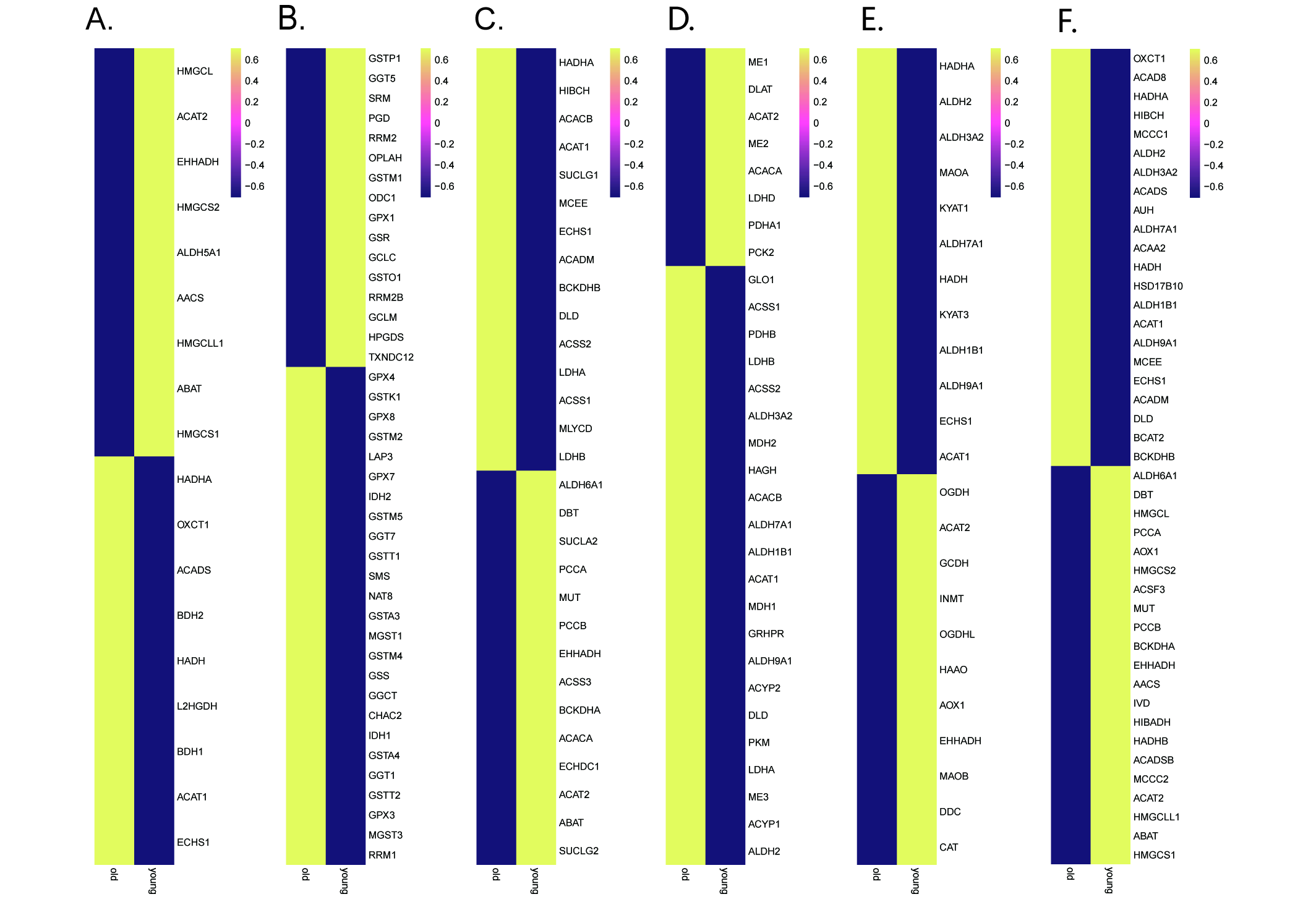

Supplement: Supplementary file 11 [file Image5.TIF]
